# The Promise of Solid Lubricants for a Sustainable Future

**DOI:** 10.1002/adma.202500867

**Published:** 2025-11-24

**Authors:** Philipp G. Grützmacher, Andrey A. Voevodin, András Korényi‐Both, Maria Clelia Righi, Christopher DellaCorte, Nazlim Bagcivan, Ali Erdemir, Carsten Gachot

**Affiliations:** ^1^ Institute for Engineering Design and Product Development Research Unit Tribology TU Wien Vienna 1060 Austria; ^2^ Department of Materials Science and Engineering University of North Texas 1155 Union Circle # 305310 Denton TX 76203‐5017 USA; ^3^ Materials and Surface Engineering Laboratories Woodward Technology Center‐2 753 Champion Way Windsor CO 80550 USA; ^4^ Department of Physics and Astronomy “Augusto Righi” University of BolognaBologna Bologna 40127 Italy; ^5^ Department of Mechanical Engineering, University of Akron Engineering Tribology Laboratory Akron OH 44325 USA; ^6^ Schaeffler Technologies AG & Co. KG Industriestraße 1‐3 91074 Herzogenaurach Germany; ^7^ J. Mike Walker ’66 Department of Mechanical Engineering Texas A&M University College Station TX 77843 USA

**Keywords:** 2D materials, friction, solid lubricants, tribofilms, wear

## Abstract

There's no question that lubricants will become more crucial in a world where technology plays an ever‐more‐important role. Lubricants save vast amounts of energy and material resources by reducing friction and increasing the lifetime of devices. It is no surprise that the lubrication industry is one of the largest in the world. The climate crisis demands solutions independent of non‐renewable fossil energy, from which most of the liquid lubricants are extracted. Solid lubricants are the focus of the research community because they provide extremely low friction with a potentially much lower environmental impact. They are used when the operating conditions of machines do not allow the use of liquids, such as in a high temperature or vacuum environment, or when contamination mitigation is critical. The future of solid lubricants is bright. Can they truly be a direct substitute for liquid lubricants in certain applications, as many researchers claim, or will they be used only for applications where operating conditions are too harsh? In this perspective, the status and prospects of several classes of solid lubricants for greener and more effective lubrication are critically reviewed, with the hope that they can help us achieve the sustainability goals for the planet.

## Graphical abstract

Lubricants are vital for technology, saving energy and resources. The industry seeks sustainable solutions beyond fossil fuels. Solid lubricants offer extremely low friction and reduce environmental impact, especially in harsh conditions. Can these solids truly replace liquid lubricants, or are they limited to extreme applications? Explore the status and prospects of solid lubricants for a greener future.

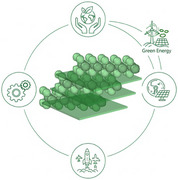

## 1 Challenges and Limitations of Solid Lubricants

**Figure 1:**
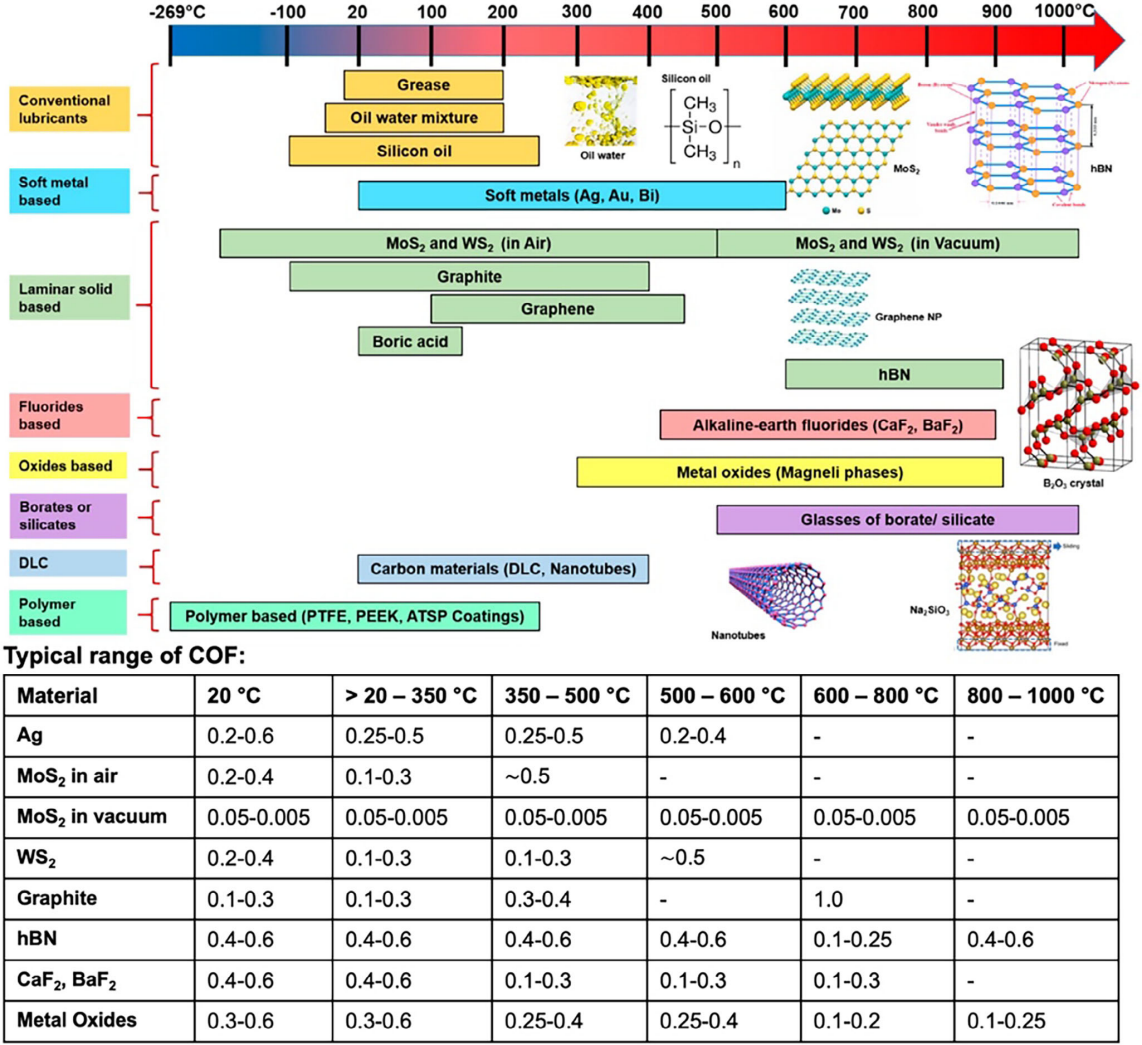
Overview chart for various lubricants and their typical operating temperatures and associated coefficients of friction. Adapted under the terms of the CC‐BY Creative Commons Attribution 4.0 International license (https://creativecommons.org/licenses/by/4.0).^[^ ^2^ ^]^ Copyright 2022, Kumar et al., published by MDPI.

Despite the use of all kinds of advanced liquid or grease lubricants, friction and wear continue to consume huge amounts of energy and consequently cause harmful emissions.^[^ ^1^ ^]^ Mainly because of increasingly demanding or extreme operating conditions, liquid lubricants are falling short in meeting expectations, especially under difficult conditions such as high vacuum, temperature extremes (cryogenic to hundreds of degrees Celsius), high speeds (or extremely low speeds), and high loads, cosmic or high radiation environments, electrified sliding interfaces, etc. **Figure** **1** illustrates this point with a notional mapping of common solid lubricant materials used and their friction coefficients on a temperature scale at the ambient pressure, where, for comparison, the operational range attainable by current hydrocarbon‐based oils and greases is also outlined.

One can see that solid lubrication today covers the entire scale of temperatures, expanding into cryogenic and high‐temperature lubrication regimes well outside of the liquid lubrication regime. Most solid lubricants can endure applications involving extreme loading or pressures, and their lubricity can be preserved over very broad ranges of temperatures, ranging from cryogenics up to >1000 °C (especially in vacuum or space environments), whereas most liquids either solidify (at low temperature) or decompose or oxidize (at high temperature). Most solid lubricants can also maintain their unique lubricity over very long periods of use and offer better industrial hygiene due to very little or no outgassing or harmful emissions. Furthermore, recent advances in research and manufacturing of nanostructured solid materials specifically tailored for friction reduction (nano‐diamond, diamond‐like carbon (DLC), inorganic fullerene‐like (IFs) nanostructures, layered 2D nanostructures, etc.) growingly demonstrate that friction coefficients well below 0.01 can be attained with solid lubrication,^[^ ^3^, ^4^, ^5^, ^6^ ^]^ providing a possible alternative to hydrodynamically assisted low friction regimes most typically achieved with oils.

Most solid lubricants have a layered or lamellar structure, which is key to their remarkable lubrication mechanisms. Specifically, when applied to a sliding contact interface, their crystalline layers undergo easy shear in a lateral direction to provide lubrication. In addition to these naturally occurring solid lubricants, there are also several other organic or manmade solids, such as hexagonal boron nitride (h‐BN), polytetrafluoroethylene (PTFE), etc., that can also provide very low friction and wear. There exist several pure or alloyed metals that can also provide low friction and wear. For example, soft metals like Ag, In, Pb, Bi, Au, and their alloys can also provide low friction by an easy shear mechanism (especially at elevated temperatures). Some solid oxides and rare‐earth fluorides can also provide low friction. Carbon‐based coatings like diamond and diamond‐like carbons are also known to be good solid lubricants. Furthermore, in recent years, a myriad of 1D, 2D, and 3D nanomaterials have been developed and shown to be very effective in minimizing friction and wear. In fact, with the use of such 2D materials like graphene, MoS_2_, h‐BN, black phosphorus, MXenes (i.e., 2D transition metal carbides, nitrides, and carbonitrides), etc., researchers have shown that friction coefficients below 0.01 (which is the superlubric sliding regime) are feasible.^[^ ^7^ ^]^

Since the industrial revolution, hydrocarbon‐based oils and greases have been addressing most of the lubricant application needs in various industries, which in the last few decades have started changing due to more strict environmental regulations and a rapidly expanding need for lubrication outside the temperature‐pressure ranges that are typical of oils and greases. The latter is not only due to the growth in space, communications, and transportation, but also commercial aerospace, nuclear power, and other industrial sectors, which are typically thought of as extreme environmental applications. For example, one rapidly growing industrial field with increasingly challenging operational conditions is e‐mobility in ground transportation, where larger bearings, and/or gears, must be lubricated at much higher speeds and loads than those found in traditional internal combustion engines (ICE).^[^ ^8^ ^]^ Additionally, as there are far fewer moving components in electric vehicles (EVs), components that have not had significant effects on the efficiency of ICE‐driven cars before are now substantially affecting the total efficiency of EVs. One of the critical components in EVs is wheel bearings. The seals for the wheel bearings run under a dry lubrication regime, and here, solid lubricants could be used to further improve EVs' efficiency. The expanding use of electrical drones for the aerial transportation of goods and services, as well as in the defense industry, is another sector that uses high‐speed mechanisms under variable loads, environments, and stringent requirements for powertrain miniaturization.

Not only for these markets but in general, the trend across almost all industries is to design more compact devices, hence leading to higher torques, loads, and temperatures.^[^ ^8^ ^]^ In combination with low‐viscosity oils to reduce frictional losses, such a situation can easily lead to reduced oil film thickness and, hence, a transition from wear‐free full‐film lubrication to mixed or even boundary lubrication regimes and thus lead to a decrease in service life.^[^ ^9^ ^]^ This is particularly relevant for EVs as they do not release combustion byproducts and therefore oils and greases must be changed less frequently, if ever (for a fill‐for‐life scenario).^[^ ^9^, ^10^ ^]^ This makes surface protection with lubricious coatings even more critical. Additionally, there are more and more electrical contacts in cars, which have to be opened and closed now and then. For such electrical contacts, solid lubricants are used to reduce friction at their opening and closing. These can be embedded into the electrical contact coating to form a self‐lubricating material.

Another rapidly growing field of application where such extreme environments prevail is the aerospace sector. The turbines used in the aerospace sector are subject to a lot of vibrations, which give rise to fretting wear.^[^ ^11^ ^]^ While there are liquid lubricants with low vapor pressure that are used for aerospace applications, such as perfluoropolyethers (PFPEs) or polyalphaolefins (PAOs) (e.g., the commercial products Fomblin or Krytox),^[^ ^12^ ^]^ they require complex sealed mechanical systems and can contaminate sensitive optical equipment due to lubricant migration out of the frictional contact^[^ ^13^, ^14^ ^]^ and therefore cannot be used everywhere. Additionally, they will either evaporate or disintegrate chemically due to UV and cosmic radiation under space conditions in the long run. Solid lubricants, on the other hand, offer numerous unique advantages in tribological applications over liquid and grease lubricants. Some of them (i.e., MoS_2_, WS_2_) work extremely well in high vacuum by providing some of the lowest friction coefficients. They do not outgas or undergo chemical or structural degradations due to cosmic rays or temperature extremes in space applications, making them perhaps the best lubricants for use in deep space explorations as well as the establishment of lunar and Martian habitats. Nevertheless, we want to stress that liquid lubricants, while sometimes inferior to some solid lubricants, are still used in space due to the experience with them as space lubricants (a term called “space heritage”). For new solid lubricant systems, flight history might be obtained by performing tribometer tests in space, such as part of the Materials on the International Space Station (MISSE) project.^[^ ^15^ ^]^ However, even with liquid lubricants in place, they might not provide complete separation (i.e., hydrodynamic lubrication) at all operation conditions or even fail due to migration or evaporation, which, for space applications, necessitates the usage of a backup lubricant. For instance, sputtered gold coatings are used for the gears that rotate the giant solar panels even though they are generally liquid lubricated. For the same reasons of reliability, solid lubricants are also employed as a backup system for mechanical safety mechanisms in high‐risk aerospace systems, such as launch vehicle abort mechanisms, which prevent activation under unsafe conditions or by unauthorized personnel. These mechanisms have to work flawlessly, and therefore, very stable legacy solid lubricants are applied. Consequently, in both these applications, the stability and reliability of solid lubricants are particularly needed.

The literature is richly populated with insightful and comprehensive papers that review key aspects of space mechanisms and the tribological challenges they entail. Early in the space program, Anderson and Glenn clearly discussed the difficulties in using liquid lubricants in space and introduced many of the solid lubricants, such as MoS_2_ and PTFE, that remain the primary solid lubricants to this day.^[^ ^16^ ^]^ In the mid‐1990s, some three decades after Anderson and Glenn's review, the US National Aeronautics and Space Administration (NASA) commissioned a “lessons learned” study that captured the state‐of‐the‐art in the liquid and solid lubrication of space mechanisms.^[^ ^17^, ^18^ ^]^ These documents reinforced earlier work by emphasizing the importance of matching the needs of the application with the capabilities of the solid (and liquid) lubricants, with respect to lifetime, friction, and environmental compatibility. Indeed, for many space tribological applications, such as deployment mechanisms, long service life is not even a requirement, but long‐term storage capability is vital. More recently, Hampson and his colleagues describe in detail an effort to design a solid lubrication “system” for a high‐temperature ball bearing of a space mechanism.^[^ ^19^ ^]^ Their “system” encompasses solid lubricant coatings, carefully selected ball and race materials, and self‐lubricating bearing cages that, together, help the bearing meet design life, bearing torque, and environmental compatibility requirements. Thus, we can see that a broad array of tribological remedies and tools (i.e., solid lubricant coatings, materials, and application approaches) have been developed and proven to meet the needs of space tribology applications.

The recent developments in new device design (such as the drivetrains of electric vehicles) not only push liquid lubricants to their physical limits but are also creating problems with sealing due to high centrifugal forces, giving rise to leakages and contamination of lubricating oils.^[^ ^20^ ^]^ These developments are certainly driving demands for more advanced innovations in lubricants that can provide higher performance under extreme operating conditions. Solid lubricants may be the only lubrication option for mitigating friction and wear‐related problems under such conditions.^[^ ^21^ ^]^ Additionally, as contamination is far less of a problem with solid lubricants, they allow for simpler designs, which is particularly relevant due to increased automation in production and assembly.^[^ ^22^ ^]^ In this context, we want to emphasize that solid lubricants are permanent, in the sense that they do not need to be pumped or brought into contact. This also reduces the complexity of the design and the number of necessary mechanical components, therefore, reducing weight, which is, for example, crucial in applications such as drones. Moreover, solid lubricants are compatible with a wide range of different materials, can improve the running‐in of slow‐moving or heavy‐duty components, prevent stick–slip and thereby noise and fretting wear (e.g., in turbines), are largely independent of speed in terms of performance, provide additional emergency lubrication under mixed or boundary lubrication regimes when used in combination with liquid lubricants, and thus facilitate maintenance‐free lifetime lubrication because they do not attract dirt or contaminating particles.^[^ ^22^, ^23^, ^24^ ^]^ Additionally, running‐in for machinery lubricated with solid lubricants happens with less wear because the surface asperities are covered with the solid lubricant, and once removed from the surface, as extremely abrasive wear particles are encapsulated by the solid lubricant film and guided out of the contact, causing less abrasive wear.

Due to the vast number of different solid lubricants, their tunability by making use of their often nanoscale nature as nano building blocks,^[^ ^25^ ^]^ possibility of mixing them with liquids and greases or creating composites of various solid lubricant materials to improve performance, allows the right solution for more and more specialized applications to be found. For instance, sputter‐deposited films of MoS_2_/Sb_2_O_3_/Au (i.e., doped MoS_2_) have been applied by physical vapor deposition (PVD) to lubricate the near‐infrared spectrometer on the James Webb Space Telescope (JWST). In this coating, there are multiple constituents that have unique design goals but still behave synergistically. MoS_2_ serves as the lubricant, while Sb_2_O3 offers load support and enhances environmental stability by creating a dense amorphous structure. This combination also improves oxidation resistance. Additionally, the gold nano‐inclusions refine the grain structure and migrate to the surface under high contact pressure, providing extra lubrication at the contact point.

The most robust solutions using solid film lubricants typically have high load support from a hard or hardened substrate, taking advantage of the “hard/soft” concept of Bowden and Tabor.^[^ ^26^ ^]^ While most liquid and grease lubricants work best with ferrous materials, solid lubricants can work with all types of materials, including some of the hardest materials to lubricate, like ceramics, Al, Ti, etc.

Unlike liquid and grease lubricants, where contamination due to their improper disposal is a crucial problem,^[^ ^27^ ^]^ most solid lubricants are also environmentally safe and recyclable. In fact, most of them (e.g., graphite, molybdenum disulfide (MoS_2_), boric acid, etc.) occur naturally and hence do not pose any safety concerns during use. Since they are available as natural mineral deposits, they also do not involve environmentally unfriendly chemical synthesis or processing methods.

Summarizing, the biggest advantages of solid lubricants in comparison to liquid lubricants lie in their temperature stability, physical and chemical stability (and therefore also less difficulties with their environmental impact), their extremely low vapor pressure, and they do not require pumping or sealing systems.

Nevertheless, the question remains whether solid lubricants are a viable alternative to liquid lubricants. It is generally best to design a machine without moving parts and if this cannot be avoided then a liquid lubricant will always provide better tribological performance than a solid lubricant. Solid lubricant coatings are applied as a design element and cannot be easily replaced or can only be replaced at great expense, in contrast to liquid lubricants, which are simply poured on the surface. What makes the situation worse is that many solid lubricant coatings may have low wear life^[^ ^28^ ^]^ which is often connected to poor adhesion of solid lubricant coatings to underlying substrates.^[^ ^24^ ^]^ Due to their finite thickness, solid lubricant films wear off gradually and hence are removed from the surface eventually. This may lead to total loss of lubricity. Therefore, prior to coating deposition, the surfaces have to be prepared by cleaning and sometimes surface roughening. In one of the author's 37 years of experience in growing functional tribological coatings and troubleshooting functional coating failures, the grand majority root cause of film adhesion failure has been consistently, with very few exceptions, based on improper pre‐coating surface preparation of the substrate to be coated. Contamination or barrier layers, by design or naturally occurring, will prevent proper adhesion of the desired surface treatment, therefore identifying the nature of what resides on the substrate surface and then the subsequent appropriate removal methodology is paramount to success. A coating can only provide an appropriate benefit if properly anchored to the substrate. Hence, surface pretreatment and monitoring of the surface's state before coating are extremely important. Additionally, many solid lubricants have variable friction coefficients based upon the environment in which they operate, this variation can be as large as an entire order of magnitude, for example, when transitioning form a terrestrial application operating in air to a space application operating in vacuum.^[^ ^29^ ^]^ Design engineers prefer to have architectures in moving mechanical assemblies that have a relatively consistent coefficient of friction, therefore a goal for tribologists is to produce solutions which are agnostic to environmental influences on the friction values. Besides, the wear lives or endurance limits may go down significantly depending on the environment. For example, MoS_2_, and WS_2_ may provide some of the lowest friction coefficients in inert gas or vacuum environments, but in humid air, their friction increases significantly, and their wear lives become very short. In the case of graphite, an opposite effect occurs, i.e., it lubricates the best in humid air but degrades very rapidly in vacuum or inert environments. One of the other major limitations of solid lubricants is their finite lifetime, especially if applied as a thin layer or coating.

Another shortcoming of lamellar solid lubricants is their poor thermal and electrical conductivity. In a high‐load and high‐speed condition where significant heat may be generated, they cannot dissipate heat fast enough from the contact interfaces, where triboelectricity may also come into play and cause electrical charge build‐up. Except for graphite, most transition metal dichalcogenides (TMDs), boron nitrides, and DLC coatings are electrically insulating to some degree, and hence, when such solid lubricants are used for friction and wear control, problems with contact electrification may occur and compromise the structural, mechanical, and tribological integrity of such interfaces. This situation will be far more important and prevalent to electric vehicle drivetrains where more intense charge builds up and hence uncontrollable discharges are very common due to stray electricity or shaft currents floating through the system.^[^ ^30^ ^]^

Certain solid lubricants are also vulnerable to aging, especially if they are subjected to high humidity, acidic gases, radiation, ambient temperature fluctuations with high frequency, or high temperatures. For example, MoS_2_ and other TMDs may undergo very slow oxidation if stored in open air and thus experience chemical/structural degradations leading to inferior lubricity. When exposed to very high temperatures, this class of solid lubricants can also oxidize or experience irreversible structural/chemical changes that can impair their lubricity. Oxidation products like MoO_3_, WO_3_ may often act as hard third‐body wear debris, thus degrading their wear lives.

Finally, a fact often neglected by the scientific community researching solid lubricants is that liquid lubricants are not only used to mitigate friction and wear, but also have crucial tasks to provide cooling, damping, and removal of wear particles from critical contact zones. While solid lubricants have shown extremely low friction and wear, these other tasks are often overlooked. As many solid lubricants are used as extremely thin coatings (<5 µm and typically <1 µm down to the nanometer range), they cannot provide these requirements.^[^ ^24^, ^31^ ^]^ Therefore, for many applications where cooling, damping, and removal of wear particles are required, solid lubricants cannot replace liquid lubricants, at least not in their pure form, but perhaps in a hybrid solid–liquid form. Finally, the functionality of a solid lubricant depends on the application, and its successful usage depends on the end user's assessment of its capabilities.

Solid lubricants are best suited for applications where they can greatly outperform liquid lubricants by friction and wear mitigation over a wide range of temperatures and pressures. We emphasize that certain constraints or requirements prevent the use of liquid lubricants, and that is when attention turns to solid lubricants. These requirements are mainly connected to thermal and chemical stability and convenience in terms of design simplicity. Solid lubricants can also be mixed with a carrier liquid that takes over the role of cooling, damping, and removing foreign particles, while the solid lubricant reduces friction and wear. Due to their outstanding tribological properties and the possibility to adapt them to any carrier liquid, these can also be environmentally friendly solutions, such as water.^[^ ^32^ ^]^ Because of the increasingly challenging operating conditions under which many mechanical systems function, the lubrication conditions may change frequently from full film lubrication to mixed lubrication regimes. Under these conditions, the surface asperities of the sliding pairs touch one another, and solid lubricant additives can be of great use by forming protective tribofilms over such asperities to reduce the intensity of asperity–asperity contacts or collisions on the surfaces. Additionally, solid lubricant coatings can be combined with oil or grease lubrication. However, it must be noted that depending on the type of solid lubricant and surface treatments, oils or greases may also reduce the service life of solid lubricant coatings by washing them away.^[^ ^33^ ^]^ An ideal example of an effective combination of solid lubricant coating and oil lubrication is DLC. To improve durability and transmission efficiency, Honda, the car manufacturer, for example, used DLC coatings in their Formula One gearboxes where gear surface slip can reach more than 20 m s^−1^ and contact pressures can be higher than 2 GPa.^[^ ^34^ ^]^ Applying a uniformly thick and hard DLC coating to the gear teeth reduced power losses by 2 kW, and in combination with an optimized oil, gearbox transmission efficiency reached 97 % while no gear‐related issues occurred during the actual races. While DLC might have had its beginnings in motorsport, it soon found its way into large‐scale industrial applications. Nowadays, it is almost present in all cars where it is used for piston rings or tappets to reduce friction and wear. In this context, communication between academia and industry should be improved, as many scientists are still doing fundamental research on Tetrahedral amorphous carbon (ta‐C) coatings, while it has already been used for industrial purposes millions of times.

In our experience, design guidelines for solid lubricants remain a problem area in industry, and often things are just “cut and pasted” from previous designs; however, there are many other things to be considered in the selection and proper design of a solid lubricant for extreme tribological performance over a wide range of application conditions and successful industrial application. The most important factors are:

‐ Proper load support.
‐ Friction and wear performance.
‐ Costs and availability.
‐ Coating architecture including constituents, microstructure, thickness, surface finish (before and after coating), etc.
‐ Limits of operating conditions (e.g., temperature‐related).
‐ Environmental degradation including corrosion, oxidation, aging, and the protection of them against such degradations thereof.
‐ Fluid and sealing compatibility.
‐ Industrial hygiene, environmental impact, and recyclability.
‐ Health, safety, toxicity.
‐ Sustainability, including their life‐cycle assessment and production‐related carbon footprints.

According to industry experts, costs are always the most important for large‐scale industrial applications, and connected to this also the possible deposition method. Materials that can be applied as paints offer cost‐effective processing and are therefore favored. Costs furthermore determine the parts for which solid lubricants can be applied. Large and heavy parts often cannot be coated economically. Additionally, the commercial availability of lubricant materials is extremely important. Materials that have shown outstanding performance like graphene cannot be produced in large enough quantities and cheap enough to be relevant for large‐scale industrial applications.

Solid lubricants have to be engineered for a certain application and they work perfectly if the requirements of the application do not exceed the capabilities of that lubricant. Since lubricants cannot be replenished, applications that have to run at high speeds for a long time exceed the capabilities of solid lubricants. However, more standardized testing is necessary to properly characterize the capabilities of new solid lubricant systems and to provide good performance data.

In terms of sustainability of some of the solid lubricant classes, such as graphite, MoS_2_, and boric acid have a great advantage in that they occur naturally and, therefore, do not require much energy for their synthesis, as it is known that the production of raw materials for lubricants is a major part of their production‐related carbon footprints.^[^ ^8^ ^]^ Nevertheless, it must be kept in mind that reducing friction has the greatest impact on the product's carbon footprint, as the energy demand due to friction greatly outweighs the energy demand and thus the resulting carbon emissions of the production process. Therefore, solid lubricants that require more energy during their synthesis but give lower friction will be useful and might even be more sustainable with respect to their emissions over their lifetime. Due to the wide range of operating conditions, there will not be a single perfect lubricant, but a variety of more and more specialized lubricants with different properties, which share properties of cleanliness, higher efficiency, durability, and are less hazardous, and cover a wider range of applications.

In terms of environmental compatibility, toxicity to the environment, and what happens to the product at the end of its useful life should be assessed first. When using chemicals that might be a health hazard, only doing research on these materials can be troublesome, and environmental concerns might come up only at the end of the life cycle of a product. So, if a solid lubricant might have a negative impact on the environment or on humans in any part of its life cycle, tribologists should try to find a better solution. One example is thin lead coatings, which work well and are much cheaper than tin or silver, but handling is toxic. Another example might be per‐ and polyfluoroalkyl substances (PFAS), which also comprise PTFE. Due to the possible health hazards of these substances, the European Commission commits to “phasing out all PFAS, allowing their use only where they are proven to be irreplaceable and essential to society” as stated in their chemical strategy.^[^ ^35^ ^]^

Nevertheless, compared to liquid lubricants, solid lubricants can be considered much more environmentally friendly and sustainable. Unlike liquid lubricants, their spillage or release to the environment does not cause life‐threatening contamination. Therefore, they hold great promise as green alternatives for future lubrication practices.

Due to their highly versatile nature, solid lubricants can be used in numerous forms. They can be prepared in very fine powder forms and dispersed in all kinds of liquids, like nano‐to‐micro scale colloids to enhance the lubrication performance of such liquids. They can also be applied as a very thin or thick layer, for example, by PVD or painting methods, to provide lubrication. Design and manufacturing self‐lubricating bulk composites using the powders, fibers, pellets, or particles of solid lubricants are also very common practices in industrial applications.

There are several factors that will push the development and application of solid lubricants. First, there is an increasing number of applications that involve the operation of mechanical equipment in harsh environments, such as space exploration, satellites, and electric vehicles. This also involves the development of advanced aerospace technologies, such as hypersonic flight systems and re‐entry vehicles, where extreme thermal environments must be managed. Second, new tribological challenges are coming up, such as equipment working in a hydrogen environment, which necessitates the development of new lubrication concepts that are compatible with such environments. Moreover, with rapidly expanding data centers around the world and the need for an increasing number of servers running continuously, solid lubricants are used very reliably inside backup generators. Additionally, the need for simpler designs and weight savings also favors the use of solid lubricants, for example, seen in drones. Finally, more stringent legal restrictions for liquid lubricants (e.g., in terms of disposal) or already existing solid lubricants (e.g., planned restricted use of PFAS in Europe) will further escalate the development of new solid lubricant systems. Concerning this, we strongly believe that these restrictions are always a chance for researchers to come up with a better solution than the already existing one.

In addition to the literature review, qualitative insights on application‐specific requirements and performance expectations for solid lubricants were obtained in this section through interviews with industry experts from the automotive and aerospace sectors.

## 2 Current and Future Research Directions

### 2.1 Hybrid Materials

One of the key advantages of solid lubricants is their ability to be incorporated into other solid materials, and thus, scientists and engineers can design a composite or hybrid system where lubrication is balanced with other application‐specific surface properties, such as hardness, mechanical strength, corrosion resistance, electrical conductivity, and other key parameters. One classical approach is the incorporation of solid lubricants into hard, load‐bearing, temperature, and oxidation‐stable matrices to generate self‐lubricating high‐strength composite materials tailored for various tribological applications. The selection of the matrix material is normally driven by requirements to surface strength, hardness, load support, fatigue resiliency, oxidation resistance, and/or other properties not attainable by solid lubricants. At the same time, the matrix will serve to protect the solid lubricant and provide for its gradual release into the tribological contact area. The current spectrum of self‐lubricating hybrid materials has expanded to multiple variants of ceramic matrices with various solid lubricant inclusions. Examples of self‐lubricating composite materials include nitride, carbide, and oxide ceramic matrices with embedded phases of graphite, dichalcogenides, and polymers, which are prepared by sintering,^[^ ^36^ ^]^ additive manufacturing,^[^ ^37^, ^38^, ^39^ ^]^ plasma and cold spraying,^[^ ^40^, ^41^, ^42^, ^43^ ^]^ PVD,^[^ ^44^ ^]^ infiltration,^[^ ^45^, ^46^ ^]^ and other methods.^[^ ^47^ ^]^ Overall, the self‐lubricating composite approach is gaining wider uses, especially for high‐ or cryogenic temperatures, under vacuum and/or corrosive environments, as well as high load and speed, such as for aerospace, forming, or marine applications.^[^ ^48^ ^]^

**Figure 2:**
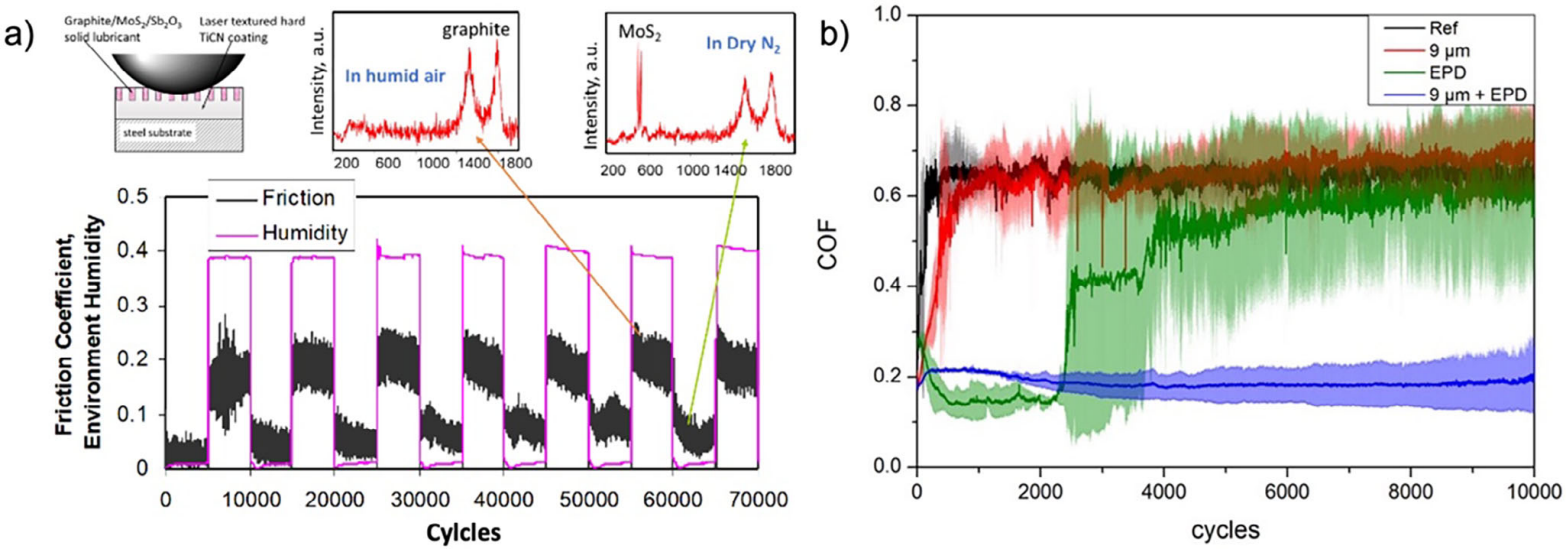
Examples of laser textured surface performance when combined with EPD lubricant applications: a) COF recovery in cycled humidity test of a laser textured TiCN coating with a burnished MoS_2_/graphite/Sb_2_O_3_ layer with insets of Raman spectra for identification of main phases present in the sliding contact at the low and high humidity cycles. Adapted with permission.^[^ ^49^ ^]^ Copyright 2025, Elsevier. b) Temporal evolution of the COF plotted as a function of sliding cycles for a polished steel reference (“Ref”), laser textured (“9 µm”), MWCNT‐coated reference (“EPD”), and finally laser textured as well as MWCNT‐coated specimen (“9 µm + EPD”). Reproduced with permission.^[^ ^50^ ^]^ Copyright 2025, Springer Nature.

Higher precision in self‐lubricated surfaces is achieved with methods allowing control over the size, shape, and distribution of solid lubricant inclusions in wear and oxidation‐resistant matrices. For example, laser surface texturing of hard ceramic surfaces to create reservoir locations, followed by filling these reservoirs, provides such precise control of the solid lubricant inclusions and allows for high precision in the selection of the location on the part surface.^[^ ^49^, ^50^ ^]^ The importance of such a choice can be for parts, where surface lubrication and mechanical strength requirements vary over the surface profile. One example is gears, where the tips and mid heights of the gear teeth need to be hard, wear‐resistant, and low friction, calling for a hard surface with added reservoirs of solid lubricants, while the teeth roots need to be fatigue resilient, calling for a less hard and highly morphologically uniform surface to prevent fatigue crack initiation. The hard surface coating control with laser texturing and solid lubricant application is hence one of the techniques for such precise location of self‐lubricating hard surface manufacturing.^[^ ^51^, ^52^ ^]^ For instance, a TiCN hard coating that features laser‐textured surface dimples and is filled with a self‐adaptive solid lubricant composite—composed of graphite, MoS_2_, and Sb_2_O_3_—demonstrates a significant ability to recover both the coefficient of friction (COF) and the primary solid lubricant phases during sliding contact. This recovery is effective across multiple cycles when transitioning from humid to dry environments in continuous sliding, as illustrated in **Figure** **2**a.

Such recovery is stable over hundreds of thousands of cycles of endurance in either of these environments,^[^ ^41^ ^]^ making such a combination of laser texturing and solid lubricants a good candidate for the lubrication of aerospace mechanisms. The dimples enhance the performance of the solid film lubricant by providing more surface area, therefore more available lubricants for wear life, and additionally a micro dimpled surface provides a 3D feature, which can trap wear debris (third bodies generated by the wear process); keeping abrasive wear debris out of the sliding contact zone and thus reduces friction and wear. Another example is the combination of carbon nanotubes deposited by electrophoretic deposition on laser‐textured steel surfaces. Reinert et al. showed the synergistic effect of both approaches using multiwall carbon nanotubes (MWCNTs) on a laser‐induced line‐like surface texture with a line spacing of ≈9 µm with a significant reduction in the COF by this combination (laser texture with 9 µm line‐spacing + an MWCNT coating done by electrophoretic deposition (EPD)) highlighted in Figure 2b as a blue line compared to the reference steel surface (Ref, 100Cr6), an MWCNT coating on the steel (done by EPD) or a solely laser‐textured surface with a line‐like texture and 9 µm line‐spacing (9 µm).^[^ ^50^ ^]^ Laser texturing allows for a large design space concerning critical features such as size and shape, and spacing of features such as dimples, grooves, swirls, and many other geometric features.^[^ ^53^ ^]^ A consideration should be that features that work the best for solid film lubricants may or may not have parity for hydrodynamic regimes. Additionally, once the best performing conditions are experimentally derived, mass finishing or super finishing techniques can provide an economical alternative to laser processing.

The approach of combining laser surface textures with solid lubricants has also been applied to MXenes, i.e., Ti_3_C_2_T*_x,_* as nanocoatings on titanium samples (Industrial standard Ti TA1) to improve the inferior tribological properties of titanium.^[^ ^54^ ^]^ In this study by Zhang et al., the authors deposited a 50 nm Ti_3_C_2_T*_x_* coating on a groove‐textured titanium surface with a resulting reduction of the COF from ≈0.57 down to 0.17. In general, the idea of combining solid lubricants such as MXenes, DLC, or black phosphorous with a textured surface offers a great deal in efficiency as the textured surface is protected by the solid lubricant on top and the textured surface provides the necessary load support, more surface area, and lubricant pockets to keep the material in a reservoir for re‐supply to the contact.

Advances in additive manufacturing (AM) extend such precise localization of the solid lubricant inclusions further, allowing for a design of even 3D architectures with application‐tailored structural strength in the bulk and self‐lubricated mechanical characteristics on the surface, thus enabling a functionally graded self‐lubricating surface design.^[^ ^55^ ^]^ One can reasonably expect further rapid growth of laser texturing, AM, and other location and size precision control methods for solid lubricant inclusions in hard, load‐supporting, and oxidation‐resilient matrices.

**Figure 3:**
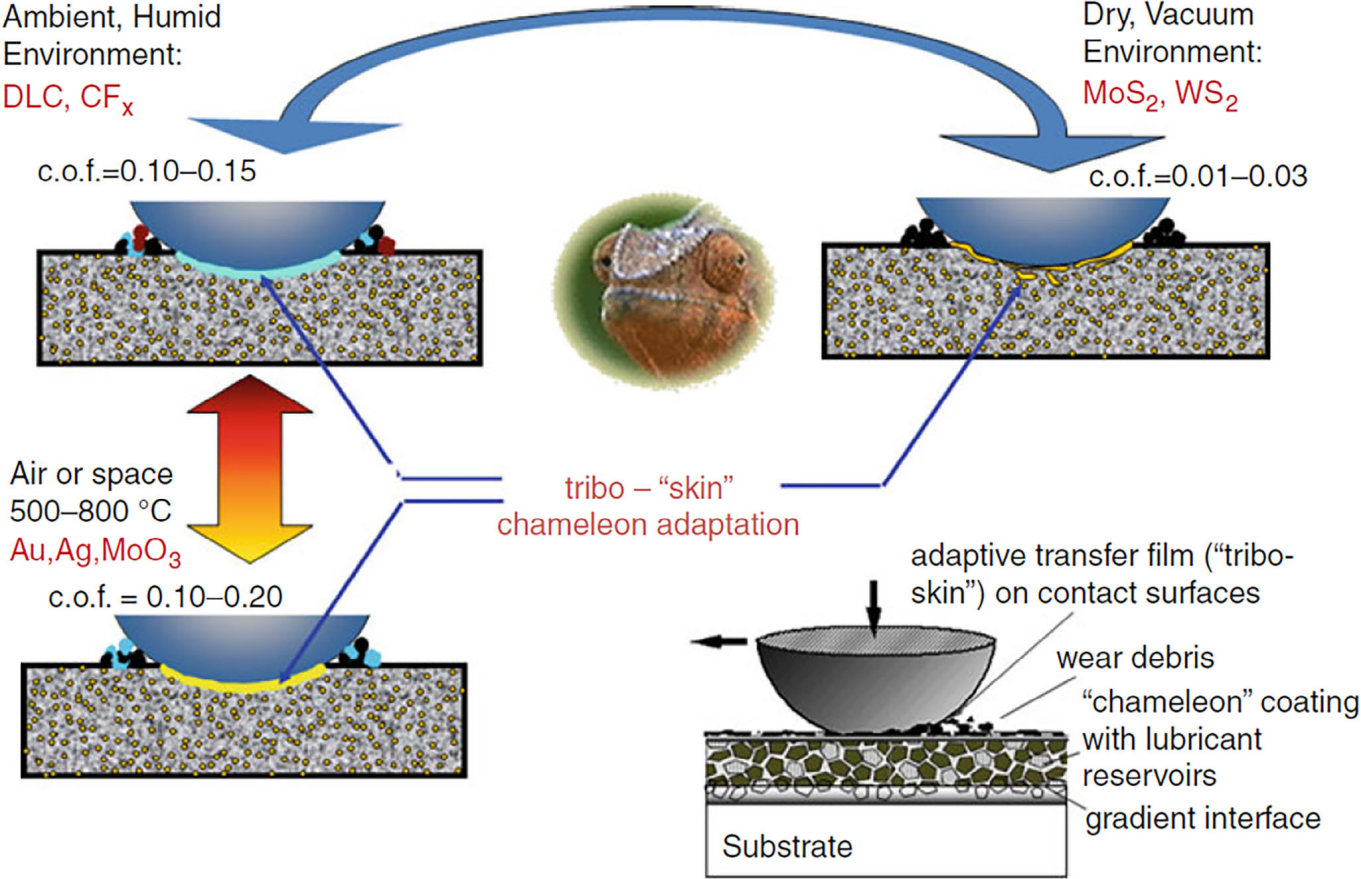
Concept of self‐adaptive behavior of “chameleon” solid lubricating coating incorporated into a hard load support ceramic matrix composite. Reproduced with permission.^[^ ^59^ ^]^ Copyright 2025, Springer.

The composite approach is also applied to solid lubricants themselves, where two or more lubricants are combined to cover a broader range of operating temperatures, pressures, and environmental conditions. One example is the previously mentioned composite solid lubricant comprised of MoS_2_, graphite, and Sb_2_O_3_. This composite solid film lubricant was found to be especially beneficial in covering a broad range of environmental humidity (Figure 2a) and pressures, ranging from space to ambient environments (i.e., utilizing MoS_2_'s ability to lubricate under dry conditions and graphite in humid environments, whereas Sb_2_O_3_ helped with strong adhesion to metallic surfaces and holding the powder lubricant together).^[^ ^56^ ^]^ This composition was adapted by the space industry, as it allows for assembling and testing sliding mechanisms on Earth and ensures their stable operation in space. Nevertheless, it had to earn space heritage as well. Graphite has long been used in early aeronautics and MoS_2_ has been used for all mechanical assemblies in vacuum requiring solid film lubrication. However, a massive failure of NASA's Galileo spacecraft, where the high‐gain antenna would not open due to degradation of MoS_2_ in a high‐humidity environment during storage, assembly, testing, and transportation, led to the need to develop solid lubricants “beyond just MoS_2_” that were more robust in variable environments. The composite of MoS_2_, graphite, and Sb_2_O_3_ was developed and gained space heritage via the MISSE space station program. The composite is still widely applied today and has been used for over 100000 different mechanisms without resulting in a single failure. The incorporation of multiple solid lubricants into ceramic matrices led to the development of several environments and temperature‐adaptive hybrid lubricating materials. For example, plasma‐sprayed powders of carbides and oxides with additions of Ag and CaF_2_ and other alkaline fluorides in the trademarked PS200 and PS300 coatings developed by NASA have found applications in propulsion components where a temperature range of operations from room temperature to ≈1000 °C was achieved by Ag lubricating at moderate temperatures and CaF_2_ at higher temperatures.^[^ ^57^, ^58^ ^]^ The adaptive approach was further expanded with PVD‐produced self‐lubricating coatings, where hard nitride, carbide, and oxide matrices were used to store inclusions of dichalcogenides (MoS_2_, WS_2_), graphite, soft metals (Ag, Au), and compounds yielding low shear strength surface oxides at higher temperatures, as reviewed previously.^[^ ^59^, ^60^ ^]^ One key approach that PVD processing allows is the incorporation of solid lubricants as nano‐inclusions, thus minimizing the reduction of the overall mechanical properties of the holding ceramic matrices and then relying on the work of friction in the contact through repeated shear and temperature spikes to form macroscopic lubricating phases covering the tribological contact. The benefit of such is the ability to cycle and reform the composition of the transfer film. This concept is illustrated in **Figure** **3** and is referred to as a “chameleon” tribological coating capable of multiple environmental and temperature cycling.^[^ ^59^, ^60^ ^]^ Overall, the hybrid lubricant approach is attractive due to the coverage of a broader range of temperatures, pressures, and mechanical loading, and gives flexibility for the processing method, sections from low‐cost surface burnishing to plasma or cold spray for thicker coatings, or a more precise surface finish via PVD coatings. These are likely to continue expanding as such methods provide precision tailoring for the balance of performance requirements under variable conditions, long endurance, and acceptable manufacturing costs.

### 2.2 Low‐Dimensional Nanostructured Solid Lubricants

In recent years, using a wide variety of nano‐manufacturing techniques, researchers have developed all kinds of low‐dimensional nanomaterials with 0D to 3D architectures.^[^ ^61^, ^62^ ^]^ Some of these are extracted from the lamellar solids (such as graphite, MoS_2_, h‐BN, etc.) mentioned earlier. Some of the examples for 0D structures include buckyballs, nano‐onions, and nano‐diamonds; for 1D structures, nanotubes, nanowhiskers, nanofibers, and nanorods; for 2D structures, graphene, h‐BN, and TMDs, black phosphorous, and MXenes; as for 3D architectures, we can include the nano/micro‐scale clusters, and powders.^[^ ^63^ ^]^ When tested for their tribological performance, most of them provided very impressive friction and wear properties.^[^ ^28^, ^64^, ^65^ ^]^ Furthermore, combinations of various low‐dimensional materials can lead to forming robust tribofilms with very low friction coefficients, as observed with MXene (Ti_3_C_2_T*_x_*)/MoS_2_ composite solid lubricant applied to steel sliding contacts.^[^ ^6^ ^]^ Further, their low‐dimensional nature provided additional benefits in terms of superior thermal and electrical conductivity, oxidation, and corrosion resistance, and even superlubricity under certain conditions.^[^ ^66^, ^67^ ^]^ Such low‐dimensional materials were also very easy to mix with liquid lubricants in colloidal dispersion and together provide superior friction and wear properties. Such low‐dimensional materials were also used in the making of self‐lubricating polymeric and other composite materials for tribological uses. Among the 0D and 1D materials, fullerene‐like MoS_2_ and WS_2_ nanomaterials have attracted special attention mainly because of their remarkable lubricity when dispersed in oils.^[^ ^68^ ^]^ Despite such attractive properties, the high cost and lack of abundant commercial availability of these nanomaterials continue to preclude them from widespread use by industry.

As far as dimensionality is concerned, metal–organic frameworks (MOFs) such as ZIF‐8, ZIF‐67,^[^ ^69^ ^]^ or COK‐47^[^ ^70^ ^]^ are an interesting class of materials as they can exist as 0D, 1D, 2D, and even 3D architectures. MOFs are crystalline coordination polymers composed of metal clusters and organic ligands.^[^ ^35^, ^36^ ^]^ The large variety of organic and inorganic building units, combined with the benefits of molecular‐level design options, makes the performance of MOFs quite interesting and impactful. Tan and Cheetham et al. reported on the structure–property relationships of hybrid framework materials and the huge potential to build up materials with tailored properties by changing secondary building units (SBUs).^[^ ^71^ ^]^

### 2.3 In Operando Formation

**Figure 4:**
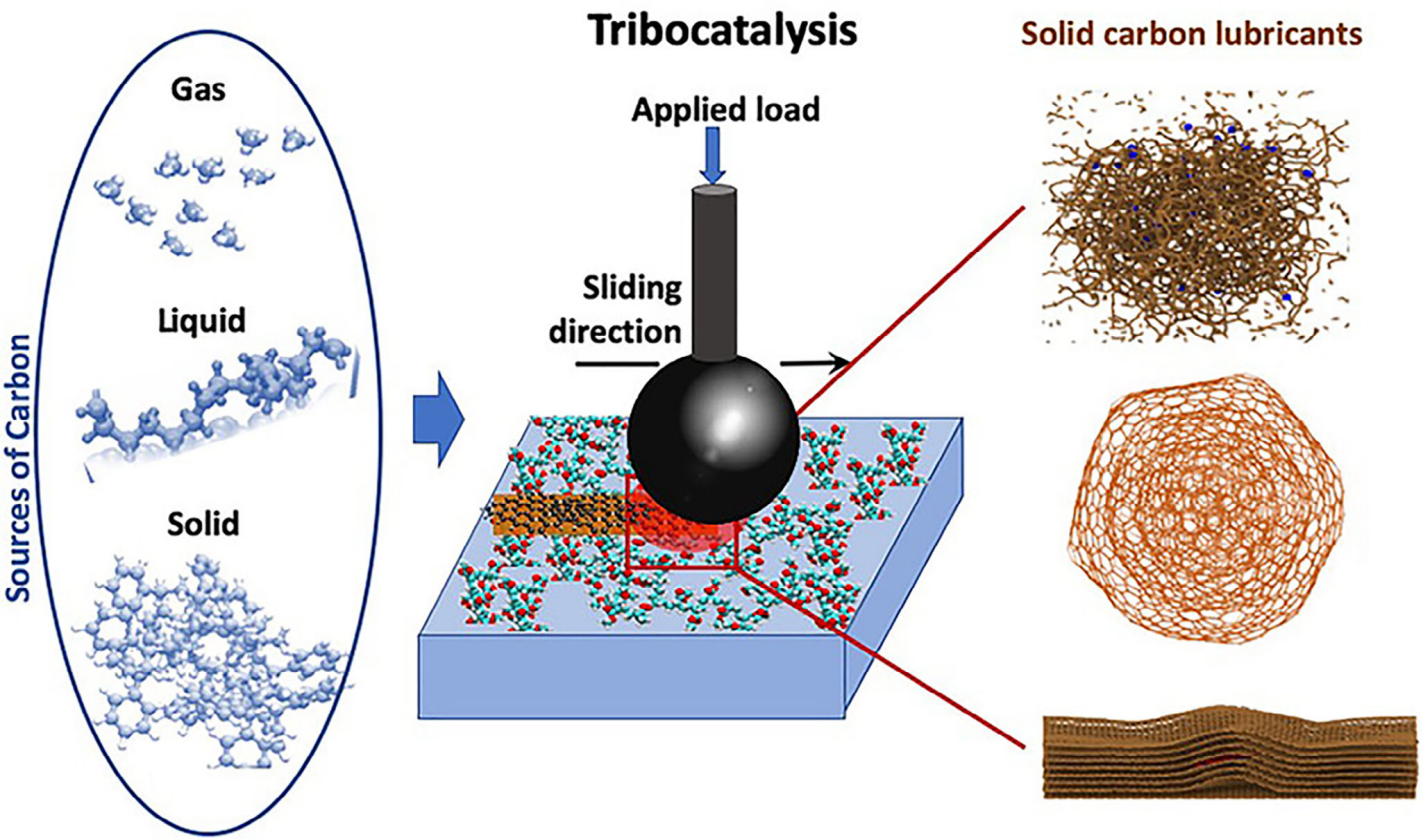
Schematic illustration of the in‐operando formation of self‐lubricating carbon nanostructures from gaseous, liquid, and solid precursors. Reproduced under the terms of the CC‐BY Creative Commons Attribution 4.0 International license (https://creativecommons.org/licenses/by/4.0).^[^ ^21^ ^]^ Copyright 2024, Lee et al., published by Frontiers Media.

The development of coating technologies and surface engineering processing methods has also opened new perspectives for adjusting surface chemistry and phase compositions for forming solid lubricants in‐operando by tribochemical and/or catalytic reactions of the contact surfaces with the environmental species and/or the counter body part. Depending on primary driving mechanisms, there are several approaches for such a self‐lubrication concept, where tribomechanical, tribocatalytic, and tribo‐oxidation processes in the contacts are probably the most widely used approaches for in‐operando solid lubricant formation. **Figure** **4** illustrates three cases for the formation of self‐lubricating nanocarbon‐based tribofilms from gaseous, liquid, and solid sources, providing ultralow friction and wear.^[^ ^5^ ^]^ There are several advantages to the in‐operando approach. The lubricant is only generated and provided to positions where high friction prevails, a minimal amount of lubricant is necessary, degradation of the lubricant in an environment different from the one prevailing during operation is avoided, and contact spots can be relubricated if necessary.

**Figure 5:**
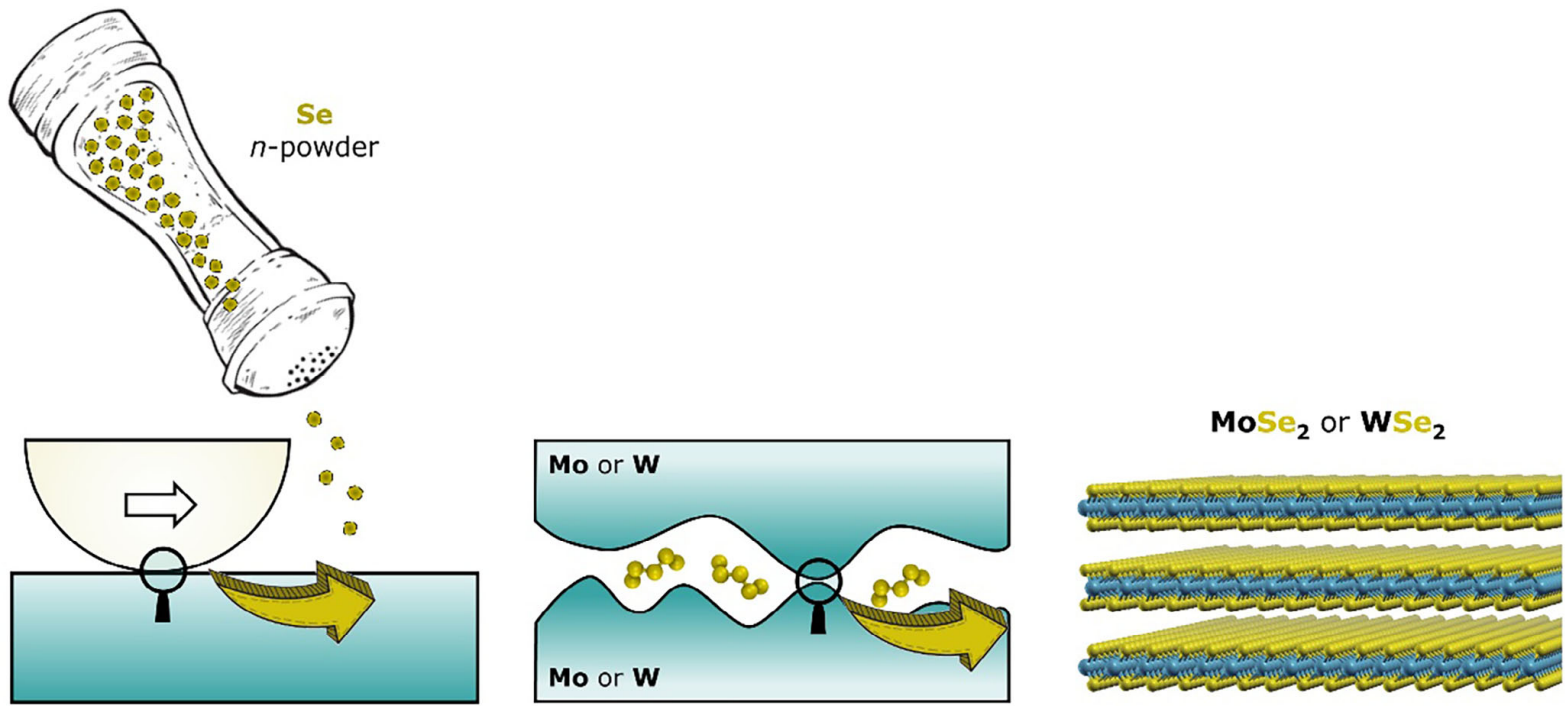
In‐operando formation of MoSe_2_ and WSe_2_ by simply using Se powder on catalytically active Mo or W surfaces. Reproduced under the terms of the CC‐BY Creative Commons Attribution 4.0 International license (https://creativecommons.org/licenses/by/4.0).^[^ ^76^ ^]^ Copyright 2023, Grützmacher et al., published by Wiley.

In other ***tribomechanical*** approaches for in‐operando lubrication, the surfaces are composed of the solid lubricant “precursor” phases, which undergo phase changes, growth, and re‐orientation to form a lubricous, easy shear surface in the contact. Examples of such approaches include amorphous or randomly oriented nanocrystalline dichalcogenide and carbon phases, which form crystalline hexagonal solids with basal planes oriented parallel to the surface in the process of repeated sliding cycles.^[^ ^72^ ^]^ These processes rely on repeated straining and local friction heat for activation and progress, and thus can equally occur in vacuum, inert, or ambient environments, as well as in fluids. This is successfully used for lubrication with initial nanocrystalline randomly oriented dichalcogenides (MoS_2_, WS_2_, MoSe_2,_ or WSe_2_, etc.) to form well‐oriented and crystalline hexagonal phases^[^ ^73^ ^]^ and a broad group of amorphous carbons, including multiple DLC variants, to use sp^3^–sp^2^ bonding rearrangements and crystallization into a graphitic phase in the contact.^[^ ^74^, ^75^ ^]^ One of the recent extensions of such approaches is the addition of the lubricant precursors not into the surfaces but into the contact environment, using oils or other liquid media as carriers, bridging liquid and solid lubrication, or even as powders in the presence of a catalytic surface consisting of Mo or W to form respective lubricous sulfides, selenides, or tellurides.^[^ ^76^ ^]^ The key advantage of the powder approach is the facilitated replenishment of the contact with a lubricous layer and the avoidance of lubricant degradation due to environmental molecules (see **Figure** **5**).

Further examples include the addition of IF nanoparticles,^[^ ^77^ ^]^ nanodiamond,^[^ ^78^ ^]^ graphene,^[^ ^79^ ^]^ ZrO_2,_ and other nanoparticles,^[^ ^80^, ^81^, ^82^ ^]^ and other precursor additives to oils or low‐viscosity hydrocarbons, which form lubricating and wear‐resistant surfaces under liquid lubrication starvation conditions.

In ***tribocatalytic*** approaches for in‐operando lubrication, the surfaces are engineered to contain catalysts that interact with the environment and facilitate the formation of lubricous phases from the environment itself. A most common and widely used example is the decomposition of hydrocarbon gases or fluids in the contact surfaces (as illustrated in Figure 4), whose composition contains catalyst metals or elements (e.g., hard nitride coatings containing Cu were shown to form a lubricous carbon‐based film in the contacts with PAO and low viscosity hydrocarbons).^[^ ^83^ ^]^ Another example of the in situ synthesis of friction and wear‐reducing carbon‐based films was presented by Xu et al., where the authors used a MoN layer with a thin 10 nm Pt film on top against a Si_3_N_4_ ball under PAO10 oil lubrication. They could prove the formation of amorphous‐carbon‐based layers by Raman spectroscopy and TEM, and showed by molecular dynamic simulations that the formation of the amorphous carbon layers was triggered by the presence of catalytically active MoN/Pt coating.^[^ ^84^ ^]^ The formation of such lubricous tribolayers was also confirmed in the presence of hydrocarbon gases, providing a 3 to 4 orders of magnitude improvement in wear rates.^[^ ^85^ ^]^ Likewise, similar films were derived from jet fuels with the use of such catalyst coatings.^[^ ^86^ ^]^ Further, it was also shown that sliding surfaces of high‐alloy steel containing transition metal catalysts like Cr, Mo, and V can also extract such carbon‐rich tribofilms from low‐viscosity lubricating oils.^[^ ^87^ ^]^ Attention, however, must be paid to the chemical analysis of tribo‐catalytically formed lubricous layers, as, particularly for Raman spectroscopy, the laser power might already be sufficient to trigger the formation of amorphous carbon layers, which are formed during the Raman analysis and are not friction‐induced.^[^ ^88^ ^]^

**Figure 6:**
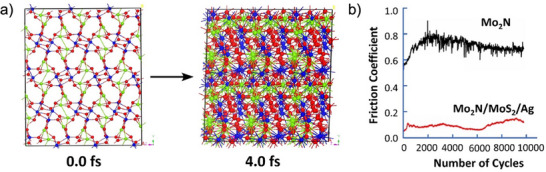
Tribooxidation triggered the formation of intrinsically layered oxides for in‐operando lubrication at elevated temperatures: a) Density functional theory–Perdew–Burke–Ernzerhof metadynamics simulations of self‐layering in Ag_2_Mo_2_O_7_ at 800 K. Reproduced with permission.^[^ ^95^ ^]^ Copyright 2025, Elsevier. b) Comparison of friction behavior for Mo_2_N and Mo_2_N/MoS_2_/Ag coatings, where the latter forms DFT‐predicted layered silver molybdenum oxide in sliding tests at 873 K in air. Reproduced with permission.^[^ ^96^ ^]^ Copyright 2025, Springer.

In ***tribo‐oxidation*** approaches for in‐operando lubrication, the combined effect of frictional heat and ambient environment temperature is used to form lubricous oxides in contact. Therefore, such phases are incorporated into the surface that have an elemental composition and reactivity, allowing for such lubricous oxide formation. Perhaps one of the most practically used examples is the addition of vanadium to the composition of hard nitride (e.g., TiAlVN) and carbo‐nitride coatings (e.g., TiVCN) designed to protect cutting tools in dry machining.^[^ ^89^, ^90^ ^]^ The high temperature of the dry machining triggers the oxidation of the coatings, thus forming VO_2_ and V_2_O_5_ phases, which have a low strength and melting point, facilitating a considerable friction reduction in the 400–700 °C range.^[^ ^90^, ^91^ ^]^ This was followed by the expansion of such coatings to include Mo‐N and W‐N coatings for MoO_3_ and WO_3_ phase self‐lubrication at above 500 °C.^[^ ^92^ ^]^ The approach of the in‐operando lubricous oxide phase formation is also beneficial for contacts designed to operate under high temperatures in ambient conditions, e.g., engines, power generators, flight controls for atmosphere re‐entry, or hypersonic vehicles, etc. Among possible self‐forming lubricous oxides, the sub‐stoichiometric Magnéli phases, such as V*_n_* O_2_ *_n_*_+1_, MoO_3_, WO_3_, TiO_2_, etc.,^[^ ^93^ ^]^ and intrinsically layered double oxides, such as AgVO_3_, Ag_2_Mo_2_O_7_, etc.,^[^ ^94^, ^95^, ^96^ ^]^ are reported for the in‐operando lubrication. The development of the new complex self‐lubricating materials is considerably enhanced by advancing computational tools, allowing them to design and simulate the tribological behavior of complex solid lubricants under various environments and temperatures. This is illustrated in **Figure** **6** on an example of a layered silver molybdenum oxide phase, which self‐forms on the surface of hard Mo_2_N coating in the presence of Ag and MoS_2_ additives in the coating composition.^[^ ^95^, ^96^ ^]^ The formation is triggered by tribochemical oxidation in air at elevated temperatures and leads to the formation of an easy‐to‐shear intrinsically layered oxide structure predicted by density functional theory (DFT) simulations (Figure 6a). Experimental verification had validated the benefit of a considerable reduction of COF at high temperatures when compared to the base Mo_2_N coating (Figure 6b). This exemplifies that the use of computational approaches will be the key in the development of future generation solid lubricants, and such approaches are discussed in Section 3 of this review.

### 2.4 Polymers

**Figure 7:**
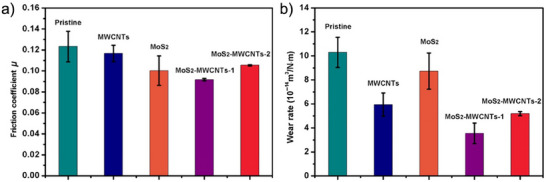
Averaged coefficient of friction in a) and wear rate b) for different filler‐reinforced polyurethane coatings. Reproduced under the terms of the CC‐BY Creative Commons Attribution 4.0 International license (https://creativecommons.org/licenses/by/4.0).^[^ ^106^ ^]^ Copyright 2018, Zhang et al., published by Springer.

Polymeric solid lubricants such as neat polymers, polymer blends, or polymer composites have been widely used to reduce friction and wear in tribologically‐challenging and extreme environments, from aerospace to biomedicine. This is mainly due to their good machineability, low density, good chemical resistance, and low COF.^[^ ^97^ ^]^ However, because of low hardness and so less durability as well as property changes in different operating environments depending on humidity and/or temperature, polymers are often used with reinforcements, fillers, or lubricants to mitigate negative effects. Sawyer et al. reported on up to 20 wt.% alumina nanoparticles (≈40 nm diameter) in compression‐molded polytetrafluoroethylene (PTFE; Teflon) with a vastly improved wear resistance.^[^ ^98^ ^]^ Since its discovery in the 1950s, PTFE has been broadly acclaimed for its low friction coefficient in sliding contact against engineering countersurfaces (*µ* ≈0.01), but its utility has been limited by its extremely high wear rate (up to *k* ≈10^−3^ mm^3^ N^−1^ m^−1^).^[^ ^98^ ^]^ PTFE—(C_2_F_4_)*_n_*—is a semi‐crystalline polymer with a melting point of ≈325 °C, which can operate up to 260 °C.^[^ ^99^ ^]^ Besides its high wear rate, PTFE also shows other drawbacks concerning thermal conductivity, high thermal expansion, low radiation stability, and relatively poor heat dissipation efficiency.^[^ ^99^ ^]^ Over the past several decades, PTFE has been used as a matrix in polymer composites that have achieved remarkable ultra‐low wear rates (*k* <10^−7^ mm^3^ N^−1^ m^−1^) while retaining moderate friction behavior (*µ* ≈0.25). Another approach to compensate for PTFE´s unfavorable wear behavior is based on polyether ether ketone (PEEK)/PTFE composites, in which the wear rate can be improved significantly while maintaining a very low COF ≈0.082 for self‐mated configurations like in a study by Van Meter et al.^[^ ^100^ ^]^ or to build up PEEK/PTFE hetero layers as described by Sun et al.^[^ ^101^ ^]^ with also ultra‐low wear rates in the range of ≈9.31 × 10^−8^ mm^3^ N^−1^ m^−1^. Apart from PTFE, polyimide (PI) also defines a high‐performance polymer with very good tribological behavior under higher contact pressures, which can be applied as coatings and thin films, being resistant to wear, corrosion, and even higher temperatures.^[^ ^102^ ^]^ The glass transition temperature is larger than 300 °C, and it can be readily used as a resin binder, for example, for self‐lubricating varnishes. In the temperature range between 25 and 100 °C, a friction transition can, however, be observed with an increase in friction and wear, which originates from the second‐order relaxation process on the molecular level of the PI. By adding either MoS_2_ or (CF)_X_, graphite fluoride, this friction transition can be entirely eliminated, and PI is usable up to 350 °C in air, for example, in foil gas bearings. Graphite‐fiber‐reinforced PI has attracted lots of attention in aircraft applications due to its high strength and high thermal conductivity.^[^ ^102^, ^103^ ^]^ Other relevant polymers, for example, to be applied as solid lubricants are ultra‐high molecular weight polyethylene (UHMWPE) in joint replacements^[^ ^104^ ^]^ as it demonstrates a very good wear resistance, especially against abrasion even in water with moderate friction, PEEK as another semi‐crystalline polymer with a high working temperature and a good chemical resistance, but also a high COF, or polyurethane (PU) exhibiting a good wear resistance in rolling contacts. In PU, there has been extensive research in the last couple of years focusing on fillers with different dimensionalities (0D, e.g., nano‐Al_2_O_3_ or nano‐SiO_2_, 1D, e.g., carbon nanotubes (CNTs) or carbon fibers, or even 2D, e.g., adding MoS_2_ or MXenes such as Ti_3_C_2_T*_x_* with significant improvements in the wear behavior by forming an effective transfer film.^[^ ^105^ ^]^ **Figure** **7** summarizes the influence of different filler materials on the COF and wear rate of PU. Adding MXenes to form polymer composites is particularly interesting, as MXenes have, by nature, a lot of active reaction sites for cross‐linking with polymers. Recent studies by Liang et al. highlighted the beneficial effect of blending Ti_3_C_2_T*_x_* MXenes (0.1, 0.5, 1, and 2 wt.%) into polyurea and polyimide copolymers remarkably improve the friction and wear depending on applied test temperatures (−100, 25, and 100 °C).

**Figure 8:**
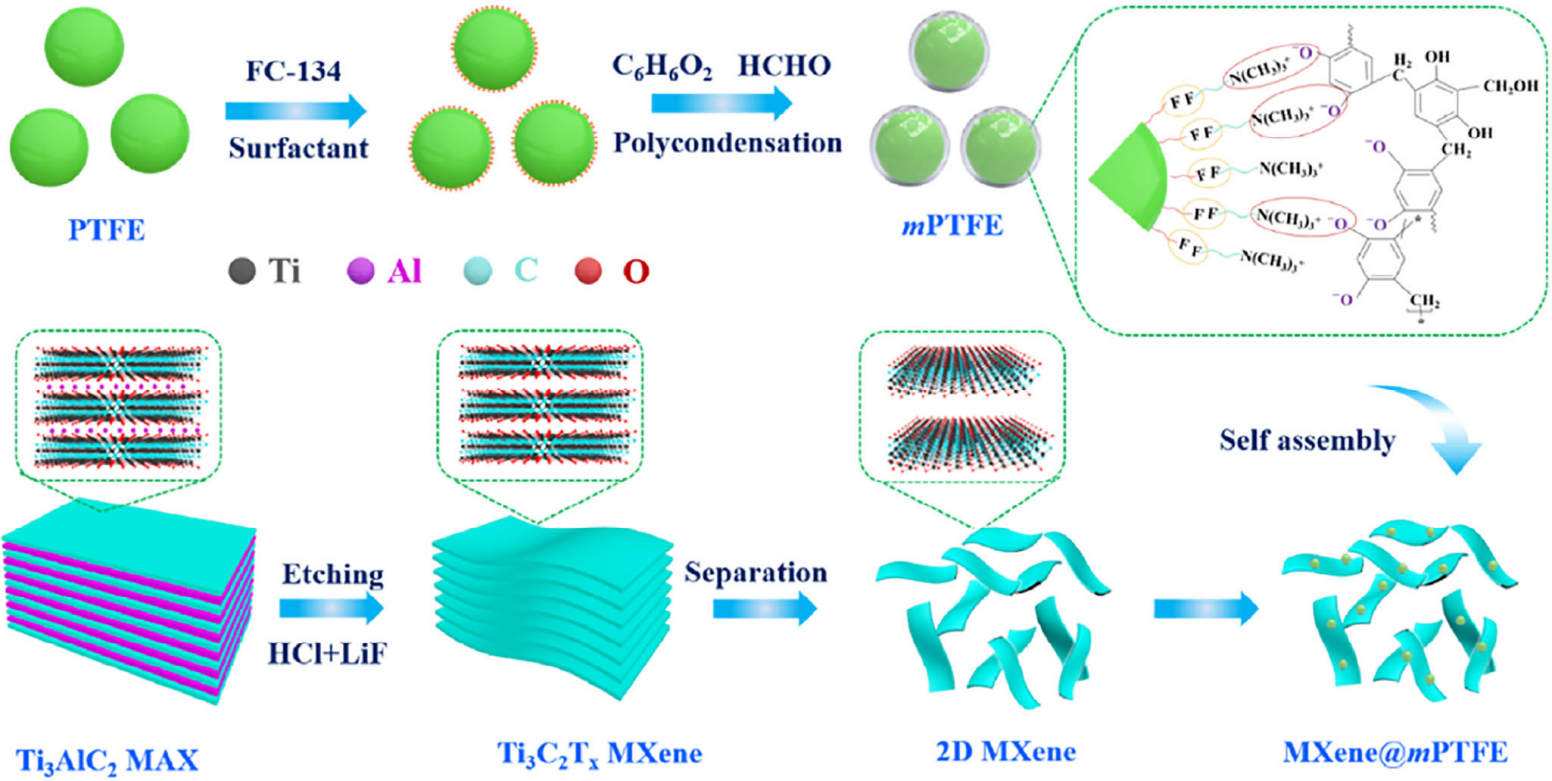
Preparation process of MXene/phenolic resin modified PTFE composites. Reproduced with permission.^[^ ^107^ ^]^ Copyright 2025, Elsevier.

Finally, results by Cui et al. revealed that the polymer composites based on phenolic resin‐modified PTFE and Ti_3_C_2_T*_x_* MXenes in PAO‐6 base oil (see **Figure** **8**) reduced the COF from 0.644 down to 0.111 and resulted in a high load‐bearing capacity of the used base oil.^[^ ^107^ ^]^

Recent and ongoing efforts have focused on developing ultra‐low‐wear solid lubricants from more sustainable materials, including melt‐processible PFA.^[^ ^108^ ^]^

## 3 Computational Insights into the Functionality, Synthesis, and Design of Solid Lubricants

A common property of solid lubricants, such as 2D materials, hydrogenated diamond/DLC, PFAS, and hydrocarbons, is their remarkable chemical stability. This property can be transferred to any substrate they adsorb onto, significantly reducing their reactivity and adhesion with any counter‐surface. Since nano‐asperity adhesion governs the macroscopic friction coefficient, as was first suggested by Bowden and Tabor,^[^ ^109^ ^]^ the principle of the function of solid lubricants is to reduce such adhesion.

**Figure 9:**
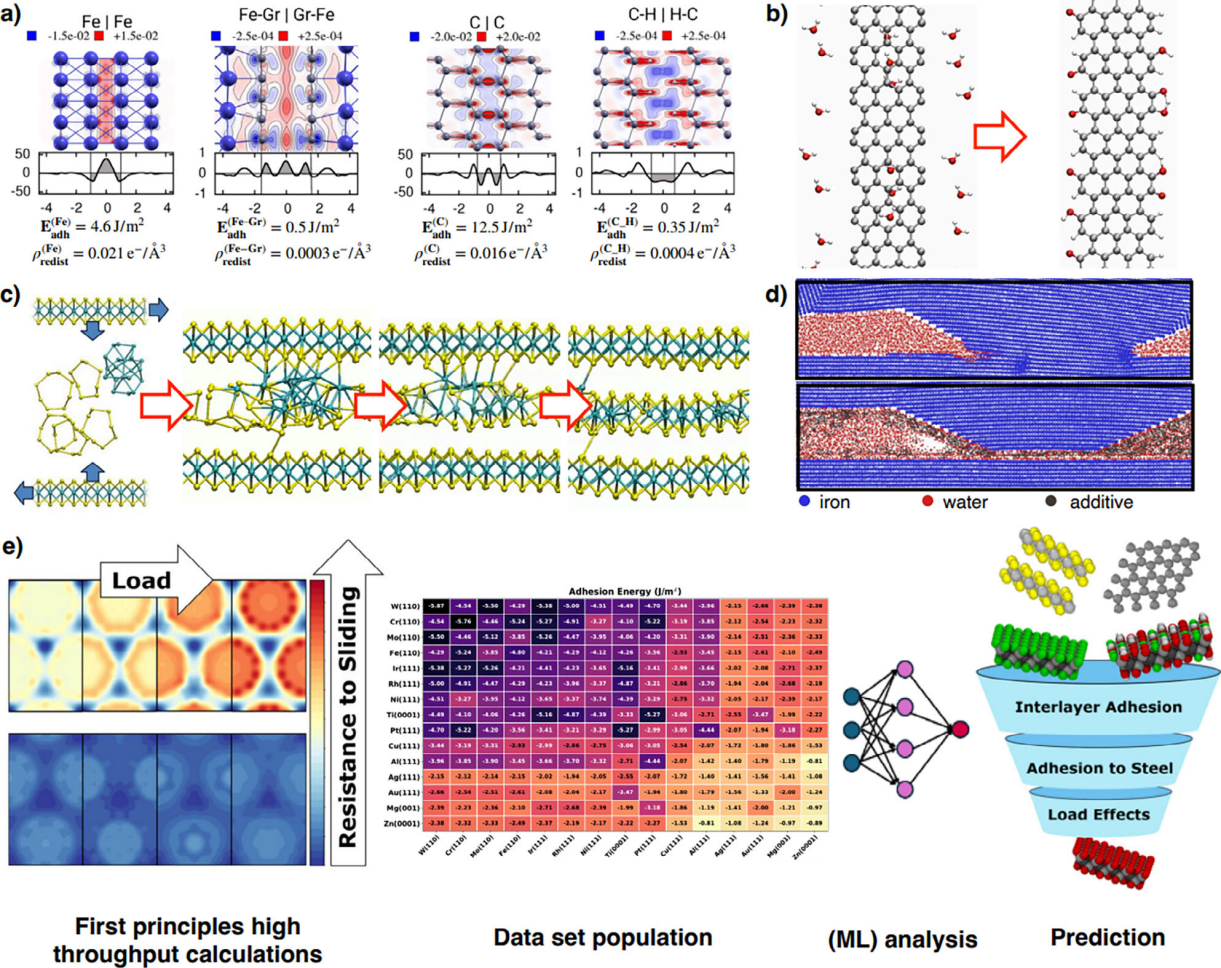
Schematic pathway from first principles calculations to prediction. e) Left/right panels: Adapted under the terms of the CC‐BY Creative Commons Attribution 4.0 International license (https://creativecommons.org/licenses/by/4.0).^[^ ^163^ ^]^ Copyright 2022, Marquis et al., published by ACS Publications. Middle panel: Adapted under the terms of the CC‐BY Creative Commons Attribution 4.0 International license (https://creativecommons.org/licenses/by/4.0).^[^ ^166^ ^]^ Copyright 2023, Restuccia et al., published by ACS Publications.

Quantum mechanical calculations have shown that the adhesion between two surfaces is directly related to the amount of electronic charge accumulated between them when brought into contact.^[^ ^110^ ^]^ This charge accumulation is high for reactive surfaces, such as native iron or diamond surfaces with dangling bonds, but can be significantly reduced by covering the surfaces with inert layers or by passivating them. **Figure** **9**a shows that graphene coverage can greatly reduce charge accumulation at iron interfaces, while hydrogen termination removes a large amount of electronic charge from diamond interfaces. These changes lead to a decrease in interfacial adhesion by two and three orders of magnitude for iron and diamond interfaces, respectively. Reduced adhesion is typically accompanied by a decrease in the corrugation of the potential energy surface (PES), which describes how adhesion varies with the relative lateral position of the two contacting surfaces. It is precisely this energy variation that gives rise to resistance forces during sliding. By analyzing the maximum resistance force along the minimum energy path—the sliding path with the highest statistical weight—it is possible to estimate the intrinsic shear strength of an interface and predict its reduction when solid lubricants are applied.^[^ ^111^ ^]^ For example, first‐principles calculations revealed that both adhesion and shear strength at iron interfaces are dramatically reduced by intercalating graphene layers or patches. These layers significantly lower the surface energy of the covered metallic regions, preventing cold welding.^[^ ^112^ ^]^ This does not imply that graphene layers must remain unbroken to function effectively as solid lubricants—contrary to what is suggested in ref.[113] where graphene's lubricity is attributed to its mechanical load‐carrying capacity rather than to the chemical effect of surface energy reduction. This latter explanation is supported by experimental data showing that even highly defective graphene layers or mere flakes can dramatically reduce the friction coefficient of steel or iron interfaces.^[^ ^114^, ^115^, ^116^ ^]^ Similarly, the dramatic reduction in the shear strength of diamond predicted by first‐principles calculations upon surface passivation with hydrogen atoms or water fragments^[^ ^117^, ^118^ ^]^ is consistent with the observed drop in the coefficient of friction (COF) of ultra‐nanocrystalline diamond in pin‐on‐disc experiments when passivating species are introduced into the tribometer.^[^ ^118^, ^119^ ^]^

It is remarkable that the reduction in shear strength predicted by first‐principles calculations often aligns with, and helps explain, the decrease in macroscale friction coefficients observed experimentally. On one hand, this confirms that interfacial adhesion plays a major role in determining the friction coefficient of solid‐on‐solid contacts, as postulated by the theory of adhesive friction;^[^ ^109^ ^]^ on the other hand, it highlights that computational approaches capable of accurately predicting nano asperity adhesion—such as those based on ab initio methods—are powerful tools for understanding the functionality of solid lubricants and guiding their optimization.

As already mentioned, the key property that makes solid lubricants effective in reducing interfacial adhesion and friction is their chemical inertness. Defects can compromise this inertness, making the material reactive, at least in the regions where defects are present. A well‐known example is graphite, which performs best in humid environments but fails to provide low friction and wear in dry or vacuum conditions.^[^ ^120^ ^]^ During tribological processes, graphite layers are easily deformed and ruptured. At high loads, graphite has also been observed to transform into turbostratic carbon.^[^ ^121^ ^]^

Ab initio calculations have shown that defects such as vacancies, edges,^[^ ^122^ ^]^ and puckers^[^ ^123^ ^]^ are significantly more reactive than the basal plane of graphite, effectively turning it from an inert medium into a chemically active one. Ab initio molecular dynamics (AIMD) simulations revealed that water molecules near these defects readily dissociate, passivating the carbon dangling bonds and thereby quenching their reactivity (Figure 9b).^[^ ^124^ ^]^ This insight helps explain why the lubricating properties of graphite are restored in humid environments.^[^ ^125^, ^126^, ^127^, ^128^, ^129^ ^]^

Similarly, AIMD simulations have enabled real‐time monitoring of the passivation mechanisms of diamond surfaces via dissociating water molecules, suggesting that load‐induced confinement promotes molecular dissociation.^[^ ^130^ ^]^ The use of silicon dopants can further enhance the rate of water splitting, leading to hydroxylated diamond surfaces with high hydrophilicity. This, in turn, facilitates the formation of a thin water film that protects the surface during tribological contact, yielding beneficial effects on friction reduction.^[^ ^131^, ^132^ ^]^ These insights from AIMD simulations helped explain experimental findings from Toyota Central R&D Labs and provided useful guidelines for designing DLC coatings with improved tribological performance for automotive applications.

DFT calculations have also shown that defects—readily formed during tribological processes—can promote the tribocharging of PTFE, a solid lubricant positioned at the bottom of the triboelectric series.^[^ ^133^ ^]^

AIMD simulations have proven to be highly effective in understanding the atomistic mechanisms involved in the in‐operando formation of solid lubricants, as described in Section 3.3. The formation of tribofilms results from complex tribochemical reactions, which are nearly impossible to monitor in real time through experiments. Typically, post‐mortem analyses provide information on the final reaction products but offer limited insight into activation mechanisms and reaction pathways. Atomistic simulations can play a crucial role in this context—particularly those based on quantum mechanical approaches, which are essential for accurately describing the enhanced reactivity conditions imposed by mechanical stress. Several general insights can be drawn by applying such methods to the study of tribologically induced formation of MoS_2_ from MoDTC molecules,^[^ ^134^, ^135^ ^]^ MoSe_2_ from Se nanopowders and Mo debris,^[^ ^76^ ^]^ graphene from CH_4_,^[^ ^85^ ^]^ and from aromatic molecules.^[^ ^136^ ^]^

The first key observation concerns the effects of load and shear. Load appears to play a major role in promoting the dissociation or deformation of chemical bonds in the precursor compounds, while shear is essential for facilitating atomic diffusion until a new stable configuration is achieved. The combined action of mechanical stress and interfacial confinement promotes structural transformations that reduce the steric hindrance of the intercalated material (Figure 9c). This explains why 2D materials or thin tribofilms are typically formed instead of 3D nanostructures. Accordingly, elements such as carbon and sulfur, which can easily change their hybridization state (e.g., from sp^3^ to sp^2^), are particularly suited for forming slippery layers under tribological conditions.

A second important aspect is the catalytic effect of the substrate, which assists molecular dissociation. For example, an ab initio comparative study on iron and iron oxide substrates^[^ ^137^ ^]^ showed that oxidation of the substrate reduces the likelihood of carbamate group detachment from MoDTC molecules, thereby delaying the formation of the MoS_2_ film. This finding is consistent with experimental observations showing a longer running‐in period before COF reduction is observed when using oxygen‐rich iron substrates in pin‐on‐disc experiments.^[^ ^138^ ^]^ Nickel, on the other hand, was found to effectively promote the tribologically induced formation of graphene from methane molecules, as demonstrated in both AIMD simulations and experiments.^[^ ^85^ ^]^ This is partly due to the good lattice match between graphene and the Ni (111) surface, which facilitates the arrangement of carbon atoms into graphene rings.

An alternative in operando pathway for synthesizing graphene is to assemble aromatic molecules, such as hypericin, which already consist of hexagonal carbon rings. In this case, the role of the substrate is critical in removing lateral groups (e.g., OH and O atoms) that saturate the molecular borders and enable solubility in aqueous media. Si‐terminated SiC surfaces have proven particularly effective in this regard. On SiC, the positively charged Si atoms—due to the higher electronegativity of carbon—can capture oxygen atoms from the molecule, creating dangling bonds that trigger polymerization, a process further facilitated by shear‐induced diffusion.^[^ ^136^ ^]^

The AIMD simulations described above have proven useful for unraveling the atomistic mechanisms behind the tribo‐formation and/or functioning of solid lubricants. However, they cannot simulate the dissolution of lubricants in a liquid medium or their subsequent confinement at microasperity contacts—conditions that mark the onset of the boundary lubrication regime, where the role of solid lubricants becomes crucial. The recent advent of machine learning potentials (MLPs) offers a promising solution to overcome these limitations. Their integration into molecular dynamics (MD) simulations enables a significant increase in the size and timescale of the systems that can be modeled, while retaining the accuracy of quantum mechanical methods. Although MLP‐based MD has been widely employed to investigate the dynamic properties of materials in various technological fields—ranging from batteries to electrocatalysis—its application to tribology remains limited to a few pioneering studies. Nevertheless, these early works have already demonstrated the great potential of ML‐MD for the design of advanced lubricants.^[^ ^137^, ^139^ ^]^ For instance, they successfully predicted the reduction in friction with increasing chain length of gallate molecules forming self‐assembled monolayers, in very good agreement with experimental observations.^[^ ^140^ ^]^ Figure 9d shows the model of an iron nano‐asperity used in large‐scale ML‐MD simulations to test the effectiveness of lubricant additives for aqueous solutions, an environmentally friendlier alternative to base oils. The onset of boundary conditions causes the cold sealing of the nano‐asperity to the substrate sliding in pure water (top panel). This catastrophic event can be avoided by including carbon‐based additives in the aqueous solution (bottom panel).

As mentioned in the previous sections, one important issue to address in improving the functionality of solid lubricants is their adhesion to substrates. Weak adhesion to the counter‐surface and strong anchoring to the substrate are often decoupled, thus smart strategies are needed to tune adhesion in opposite directions. Experimentally testing a large number of compounds and surface modifications to achieve this dual behavior can be prohibitively time‐consuming and costly. However, the exponential growth of computing power and the development of efficient data mining and curation techniques based on machine learning have opened new avenues through computational high‐throughput (HT) materials discovery.^[^ ^141^, ^142^ ^]^ This approach—based on the parallel, automated screening of hundreds of materials—has recently been adopted to advance various material‐based technologies, including catalysis, electronic devices, magnetic storage, Li‐ion batteries, thermoelectrics, superconductors, and high‐entropy alloys.^[^ ^143^, ^144^, ^145^ ^]^ These databases not only provide high‐level results but also grant access to raw data, enabling further analyses and comparisons. Manual analysis is infeasible due to the vast quantity of data generated during high‐throughput screenings. Therefore, ML tools are widely used to mine and classify the data and to guide the expansion of databases in promising directions (Figure 9e). The use of ML in tribology has recently been reviewed in several perspective articles.^[^ ^146^, ^147^, ^148^ ^]^

In a recent HT study, 1475 2D materials were screened to identify potential solid lubricants. A geometry‐independent lubricating figure of merit was introduced, and several low‐friction candidates were identified, providing a standardized metric for assessing superlubricity.^[^ ^149^ ^]^ Machine learning approaches have proven especially effective in reducing the computational effort associated with HT calculations, as demonstrated in recent reviews on 2D materials for various technologies.^[^ ^150^ ^]^ Specifically in the search for solid lubricants, ML‐assisted HT calculations have been used to screen millions of van der Waals heterostructures, leading to the creation of a database of promising 2D materials for friction reduction.^[^ ^151^ ^]^ Other researchers employed a molecular simulation design framework to conduct a combinatorial study of 9747 unique films, applying an ML algorithm to evaluate how terminal group chemistry influences tribological performance.^[^ ^152^ ^]^ Fronzi et al. developed a machine learning framework to predict interlayer binding energies and elastic constants across a vast space of 18 million van der Waals heterostructures. This approach drastically reduces computational costs compared to traditional DFT calculations and has been instrumental in identifying promising 2D superlubricant candidates.^[^ ^151^ ^]^

The HT DFT/ML calculations described above mainly focus on the interlayer sliding properties of 2D materials. Applying HT screening to evaluate the adhesion of 2D layers on solid substrates is a more challenging task, as it requires lattice matching between different materials. Recent computational studies—though not specific to tribology—have addressed this challenge, as reviewed in ref.[153] A new method has been proposed for predicting the atomic structure of 2D materials on arbitrary substrates, combining an evolutionary algorithm, lattice‐matching techniques, automated training of ML interatomic potentials, and ab initio thermodynamics.^[^ ^154^ ^]^ In another approach, Boland and Singh developed an open‐source high‐throughput workflow to computationally search for substrate surfaces with low lattice mismatch for arbitrary 2D materials, and to calculate interface interaction energies using van der Waals‐corrected DFT. This framework enables the prediction of stable 2D/substrate heterostructures and the analysis of substrate‐induced structural changes and charge doping.^[^ ^155^ ^]^

A high‐throughput (HT) software**, TribChem**,^[^ ^156^ ^]^ specifically designed for calculating the tribological properties of solid interfaces—namely adhesion and intrinsic shear strength—was recently released^[^ ^157^ ^]^ and applied to screen these properties across a wide range of interfaces.^[^ ^158^ ^]^ The first application focused on homogeneous interfaces formed by matching two equivalent surfaces of elemental crystals.^[^ ^110^ ^]^ From the resulting database, general trends emerged, such as the existence of a power‐law relationship between interfacial adhesion and shear strength,^[^ ^156^ ^]^ and the identification of their fundamental origin in the electronic charge distribution at the interface.^[^ ^159^ ^]^ A comprehensive database was built with the adhesion values of hundreds of metal pairs, and an analytical expression for predicting interface adhesion from the properties of the constituent materials—such as the surface energies of the mated interfaces—was derived using the machine learning algorithm **Sure Independence Screening and Sparsifying Operator (SISSO)**. This powerful data‐driven method enables accurate predictions in the form of interpretable equations.^[^ ^160^ ^]^ Moreover, it was demonstrated that adhesion can be tuned through targeted chemical surface modifications. By screening the effects of different adatoms adsorbed on one of the two surfaces, certain chemical species—such as sulfur, phosphorus, and in particular fluorine—were found to strongly reduce the adhesion not only of metallic interfaces,^[^ ^161^ ^]^ but also of non‐metallic ones.^[^ ^162^ ^]^ Conversely, other elements like boron were shown to act as adhesion enhancers. This understanding is relevant for designing new solid lubricants or improving existing ones. For example, a recent study on **MXenes** revealed that interlayer adhesion is governed by the chemical composition of the outermost atomic layer, with fluorine‐terminated surfaces being the most lubricous.^[^ ^163^ ^]^ It was also shown that defects—such as isolated or clustered vacancies and edges—significantly affect surface hydrophilicity,^[^ ^164^ ^]^ potentially leading to dramatic changes in lubricating performance depending on air humidity, as observed for **MoS_2_** and **graphene**.^[^ ^124^, ^165^ ^]^ These findings suggest that a future HT screening of the effects of defects in solid lubricants would be highly beneficial.

## 4 Synthesis and Circularity/Life Cycle Assessment

Despite the huge scientific and technological importance of solid lubricants, which are often based on nanomaterials, they also pose a potential risk for both the surrounding environment and human health (for example PTFE/PFAS). To assess the environmental performance of a product or material from material extraction to disposal at end‐of‐life, life cycle assessment (LCA) can be used.^[^ ^167^ ^]^

LCA of solid lubricants such as TMDs evaluates the environmental impacts throughout the entire life cycle of these materials, from extraction and synthesis to application and disposal. One advantage that solid film lubricants hold on the disposal side of the life cycle is their innate lack of thickness (typically, 1–5 µm) and therefore low relative volume percent presence on a bulk material at its end of life. TMDs, like molybdenum disulfide (MoS_2_) and tungsten disulfide (WS_2_), are particularly valued for their excellent tribological properties as solid lubricants. The production of TMDs typically starts with the mining and extraction of transition metals and chalcogens. These processes pose significant environmental challenges, including habitat destruction, energy consumption, and pollution. Therefore, it is crucial to understand the sustainability of the raw materials used in TMD synthesis, considering aspects such as the environmental costs associated with extraction, as well as the recycling potential of the materials. Synthesis techniques for TMDs, including chemical vapor deposition (CVD), mechanical exfoliation, and chemical exfoliation, each have their specific energy requirements and waste generation profiles. The energy used during these synthesis methods greatly affects the overall environmental impact, particularly fossil fuels fuel energy consumption. It is essential to evaluate the efficiency, lifespan, and performance of TMDs in various applications, such as transistors, sensors, and energy storage, to determine their environmental advantages over alternative materials. Moreover, understanding how TMDs can be integrated into existing technologies, along with their recycling potential and disposal implications, is critical. The end‐of‐life management of TMDs, including landfill and incineration practices, can substantially influence the overall LCA results, especially considering possible hazards related to heavy metals. The feasibility of recycling TMDs or recovering valuable materials at the end of their life cycle can reduce negative environmental impacts. LCA typically assesses several environmental impact categories, which include:

‐ Global warming potential (GWP)
‐ Energy use
‐ Water use
‐ Toxicity (both human and ecological)
‐ Resource depletion

Familiarity with regulations governing the mining, manufacturing, and disposal of TMDs is also important. Lifecycle impacts may shift due to market dynamics, which could affect material selection and manufacturing processes. Conducting an LCA for TMDs is a complex endeavor, and it calls for a holistic approach to fully grasp their environmental impacts. Ongoing research and advancements in synthesis methods, recycling technologies, and sustainable practices are expected to improve the sustainability profile of TMDs in the future. As interest in TMDs rises, especially in advanced applications related to electronics and energy, comprehensive and responsible LCAs will be increasingly important for shaping sustainable development strategies.

Hachhach et al. performed LCA (cradle‐to‐gate approach) of large‐scale production of MoS_2_ nanomaterials, considering a functional unit of 1 kg of MoS_2_.^[^ ^167^ ^]^ The production of MoS_2_ consumes many resources and energy, relating to global warming and high land occupation. More precisely, the process results in a release of 391.33 kg of CO_2_. Nevertheless, the impact due to toxicity is negligible for humans, flora, and fauna. Additionally, by improving the production process, the environmental impact can be reduced. Substituting LiOH, which is used as a pH regulator, by NaOH the process becomes cheaper and cleaner, resulting in a 56% decrease in the consumption of non‐renewable energy and land occupation. However, this does not have a positive impact on the release of CO_2_ because this originates mainly from molybdenum production.

**Figure 10:**
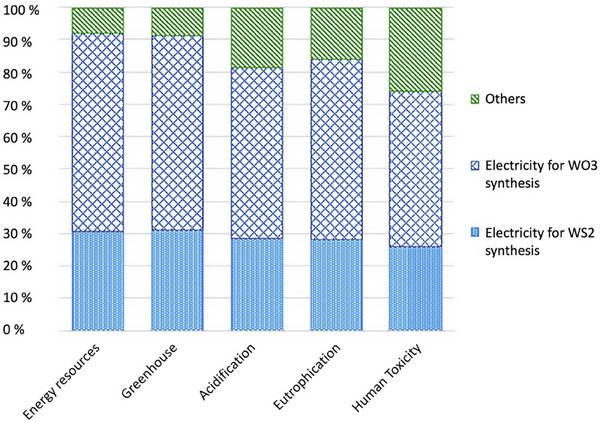
Electricity demand to produce 1 g of nanosized WS_2_ compared to the synthesis of WO_3_ as a precursor, differentiated in energy resources, Greenhouse contribution, acidification, eutrophication, and human toxicity. Reproduced with permission.^[^ ^168^ ^]^ Copyright 2025, Elsevier.

Another example is WS_2_, which can be utilized as a highly effective solid lubricant and an additive for liquid lubricants. Research by Bobba et al^[^ ^168^ ^]^ illustrates that the overall environmental impact primarily stems from energy consumption during both the synthesis and manufacturing processes of WO_3_. **Figure** **10** shows the electricity contribution during the synthesis of 1 g of nanosized WS_2_.

**Figure 11:**
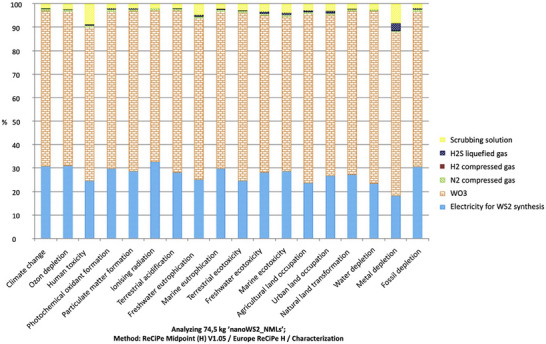
Detailed environmental impact contributions of 1 g of nanosized WS_2_ produced by the ReCiPe2008 method. Reproduced with permission.^[^ ^168^ ^]^ Copyright 2025, Elsevier.

**Figure** **11** shows a more detailed environmental impact of the production of 1 g of nanosized WS_2_ by the ReCiPe method 2008, which is a method for the impact assessment in an LCA analysis. The production of WO_3_ is the most critical step. Electricity usage is never below 26%, and in summary, the synthesis of WS_2_ and the production of WO_3_, as the primary step, is very energy‐intensive.^[^ ^140^ ^]^

Another interesting example is MXenes. They have become critically important for a range of applications since their discovery. However, their LCA remains insufficiently explored. Dadashi Firouzjaei et al. conducted a comprehensive “cradle to gate” LCA that decisively evaluates the cumulative energy demand (CED) and environmental impacts associated with the laboratory‐scale synthesis of Ti_3_C_2_T*_x_*, the most widely studied MXene composition.^[^ ^169^ ^]^ The results are clear: laboratory electricity consumption during synthesis accounts for more than 70% of the environmental impacts. To put this into perspective, while manufacturing 1.0 kg of industrial‐scale aluminum generates ≈23.0 kg of CO_2,_ and copper foil produces ≈8.75 kg, the synthesis of 1.0 kg of Ti_3_C_2_T*_x_* in the lab leads to a staggering release of 428.10 kg of CO_2_ and, therefore, a little more than the production of MoS_2_. Electricity usage is the dominant factor contributing to the environmental footprint of Ti_3_C_2_T*_x_* MXene synthesis. The limited role of chemical usage reinforces the necessity of leveraging recycled materials and renewable energy to significantly enhance the sustainability of MXene synthesis. Understanding the LCA of MXenes is essential for their successful industrialization. The production of 1 kg of Ti_3_C_2_T*_x_* MXene demands roughly 15 to 33 times more energy than the equivalent amount of copper and aluminum foil, respectively. A powerful strategy for reducing energy consumption, costs, and environmental impacts in the synthesis of Ti_3_C_2_T*_x_* MXene is to employ more affordable and recycled A‐layer and carbon sources for the X‐layer. A‐layer elements can be readily sourced at reasonable prices from metal scraps; for example, an astonishing 33 million tons of aluminum scrap available worldwide. Notably, recycled aluminum not only offers a cost advantage compared to bauxite ore but also significantly lowers greenhouse gas emissions. This approach will undoubtedly lead to a more sustainable future for MXenes.

We identified two critical challenges in applying LCA from the literature review.

- The urgent need for implementing robust datasets and…
- It is imperative to improve communication among diverse sectors involved in nanotechnology, as well as between manufacturers and consumers.

Solid lubricants can reduce maintenance costs and energy losses during operation. Compared to liquid lubricants, they are less critical in terms of spills and disposal, as large quantities are required for liquid lubricants, while only nanometer‐thin layers are often sufficient for solid lubricants. They can also contribute to downsizing, which leads to lower energy and material consumption. Nevertheless, their sustainability profile is influenced by raw material sourcing, manufacturing, and end‐of‐life challenges. Many high‐performance solid lubricants, such as molybdenum disulfide, tungsten disulfide, and hexagonal boron nitride, rely on finite mineral resources with energy‐intensive extraction processes. Advanced coating techniques, including chemical vapor deposition and sputtering, can require high energy input and hazardous chemicals. During service, wear particles may pose occupational health risks, and worn coatings are generally difficult to recycle or regenerate. At the end‐of‐life, components with solid lubricant coatings often require disposal via specialized waste streams due to the presence of metals or persistent fluoropolymers. Consequently, the overall environmental benefit of solid lubricants depends on a full life cycle assessment that balances production impacts against operational savings.

## 5 Concluding Remarks

This Perspective demonstrates that solid lubricants are already well represented and hold further great promise for their expanded use in applications involving extreme conditions and environments. Compared to their liquid counterparts, some solids provide extremely low friction coefficients and wear rates, which are much needed for reducing energy and carbon intensities of moving mechanical systems. They are also the type of lubricants suitable for use in high vacuum, temperature extremes (from cryogenic to hundreds of degrees Celsius), or at high speeds and loads in cosmic or radiation environments, electrified sliding interfaces, etc. Due to environmental concerns, there is an increased need to reduce the use of fossil or petroleum‐derived lubricants, which are also unsuitable for cryogenic and high‐temperature lubrication regimes. Fortunately, there exist several new solid lubricants (i.e., DLC, graphene, and other 2D materials) in addition to their traditional analogs that can either complement or reduce the use of liquid lubricants in support of global sustainability goals. In particular, DLC coatings are now used in a wide range of industrial applications due to their ultra‐low friction and wear under both dry and lubricated conditions.

For applications involving extreme conditions, a hybrid lubrication approach (i.e., combined uses of solid and liquid lubricants in colloidal or mixed media) can be the most appropriate approach for broad ranges of temperatures and environments. Unfortunately, some limitations on the uses of traditional solid lubricants (especially the sensitivity of metal dichalcogenides, graphite, and boric acid, to relative humidity) still persist. Through alloying and/or hybridization, such sensitivity can be curtailed somewhat but the other limitations related to temperature extremes persist and present continued challenges. Some progress has been made in the design and fabrication of oxide‐based solid lubricants to circumvent limitations on high‐temperature lubrication needs, but further research would be needed to overcome those remaining challenges in the development of self‐lubricating/self‐healing oxide‐based solid lubrication approaches so that long‐lasting and highly effective lubrication of gas turbines (especially gas foil bearings, hot metal forming or forging, etc.) can be achieved.

Most solid lubricants (i.e., DLC, MoS_2,_ etc.) are applied to tribological surfaces as thin solid films by simple gas‐driven impingement, mechanical burnishing, PVD, and CVD methods. Bulk or thick layers produced by a variety of spray methods, such as cold and/or plasma spraying, or functionally graded layers produced by additive manufacturing, are also available and could be highly desirable for long‐lasting lubrication needs. Solid lubricants can also be blended or mixed with metallic, ceramic, and polymeric materials to provide self‐lubricating composites. Most of the polymeric seals used in gas compressors and retainers or cages in ball and roller bearings use such composite materials. Mechanistically, a solid‐lubricant‐rich transfer film forms on the sliding surfaces, and such a film ensures low friction and long wear lives during sliding. As for thin or thick film coatings made of solid lubricants, one must make sure of strong bonding or adhesion to substrate materials for long service lives. Luckily, most of the PVD and CVD methods are capable of providing very good adhesion between such coatings and the substrate materials. Most often, a bond or transition layer is used to further enhance film adhesion and hence durability.

Some of the emerging applications, such as electric vehicle drivetrains, require high thermal and electrical conductivities at their moving contacts to safely dissipate heat and discharge currents. For such situations, solid lubricants with high thermal and electrical conductivities (such as noble metals) can be considered. Combining high thermal and electrical conductivity with high chemical inertness and low shear properties can be very useful for such applications. Silver has already been used for the lubrication of rotating anode X‐ray tubes of CT scans and magnetic resonance imaging machines in the medical field. Due to their excellent conductivity, some of the low‐dimensional materials, like graphene, may also be a good candidate for such applications. With growing interest in deep space explorations and the establishment of permanent habitats on the faces of the Moon and Mars, solid lubricants can also play important roles in fulfilling safe and long‐lasting mobility needs of rovers and robots alike, not to mention myriad other mechanisms that make such long missions possible.

As mentioned, lubrication of moving surfaces at high temperatures remains a challenge. At such temperatures, most layered solid lubricants either fail or provide very limited and short‐duration lubrication. A suit of self‐lubricating composite lubricants developed by NASA engineers since the late 1970s (i.e., PS 200, 300, and 400 series) remains one of the most effective for applications (i.e., gas foil bearings, etc.) involving high temperatures. Further, the recent developments in the design and fabrication of alloys and/or thin/thick coatings that can form lubricious oxides in situ also hold great promise for high‐temperature applications. To solve the problem of non‐reversibility of many high‐temperature solid film lubricants if cycled back to lower temperatures, a series of chameleon‐type adaptive lubrication strategies that can quickly respond to changes in thermal and environmental constraints was also shown to be highly effective in controlling friction and wear over a broad range of temperatures and environments.

As applications become increasingly complex and operating conditions harsher (e.g., electric vehicles, space, hypersonics, nuclear power), more specialized lubricants are needed. Here, solid lubricants that offer high tunability might be advantageous, such as MXenes, MOFs, or hybrid systems. Furthermore, with the help of High‐Throughput Material Discovery, such lubricant solutions, adapted for specific, highly demanding applications, can be developed in a timely fashion.

Briefly, solid lubricants have much to offer for our global sustainability goals. Most of them are naturally occurring, hence environmentally safe. They will undoubtedly continue to play critical roles in the smooth and safe operations of all kinds of moving mechanical systems on our planet and beyond.

## Conflict of Interest

The authors declare no conflict of interest.

## Author Contributions

P.G.G. and C.G. developed the overall idea of the perspective article and contributed to writing the main parts of the paper, did editing and visualization. A.E., A.K.‐B., A.A.V., M.C.R., N.B., and C.D. contributed to the general article structure, writing, and proof‐reading.

## Data Availability

The data that support the findings of this study are available from the corresponding authors upon request.
